# A Novel Rat Model of ADHD-like Hyperactivity/Impulsivity after Delayed Reward Has Selective Loss of Dopaminergic Neurons in the Right Ventral Tegmental Area

**DOI:** 10.3390/ijms241411252

**Published:** 2023-07-08

**Authors:** Sarah E. Kohe, Emma K. Gowing, Steve Seo, Dorothy E. Oorschot

**Affiliations:** Department of Anatomy, School of Biomedical Sciences, Brain Health Research Centre, University of Otago, Dunedin 9016, New Zealand

**Keywords:** ADHD hyperactive/impulsive presentation, ventral tegmental area, midbrain dopaminergic neurons, fixed-interval extinction test, open field test, stereology

## Abstract

In attention deficit hyperactivity disorder (ADHD), hyperactivity and impulsivity occur in response to *delayed* reward. Herein we report a novel animal model in which male Sprague-Dawley rats exposed to repeated hypoxic brain injury during the equivalent of extreme prematurity were ADHD-like hyperactive/impulsive in response to delayed reward and attentive at 3 months of age. Thus, a unique animal model of one of the presentations/subtypes of ADHD was discovered. An additional finding is that the repeated hypoxia rats were not hyperactive in the widely used open field test, which is not ADHD specific. Hence, it is recommended that ADHD-like hyperactivity and ADHD-like impulsivity, specifically in response to delayed reward, be a primary component in the design of future experiments that characterize potential animal models of ADHD, replacing open field testing of hyperactivity. Unknown is whether death and/or activity of midbrain dopaminergic neurons contributed to the ADHD-like hyperactivity/impulsivity detected after delayed reward. Hence, we stereologically measured the absolute number of dopaminergic neurons in four midbrain subregions and the average somal/nuclear volume of those neurons. Repeated hypoxia rats had a significant specific loss of dopaminergic neurons in the right ventral tegmental area (VTA) at 2 weeks of age and 18 months of age, providing new evidence of a site of pathology. No dopaminergic neuronal loss occurred in three other midbrain regions. Fewer VTA dopaminergic neurons correlated with increased ADHD-like hyperactivity and impulsivity. Novel early intervention therapies to rescue VTA dopaminergic neurons and potentially prevent ADHD-like hyperactivity/impulsivity can now be investigated.

## 1. Introduction

The objectives of this research were, first, to further characterize behaviour in a rat model of ADHD-like hyperactivity in response to delayed reward. Specifically, we measured ADHD-like impulsivity in response to delayed reward and re-checked in a new cohort whether the animals were hyperactive and impulsive to delayed reward and attentive. Our aim was to establish whether we had discovered a new animal model of the ADHD-like hyperactive/impulsive presentation in response to delayed reward. A second objective was to characterize locomotor behaviour in an open field apparatus since this is an extensively used test, but it does not measure the hyperactivity in response to delayed reward that is characteristic of ADHD (see further details below). ADHD is linked to deficits in the midbrain dopaminergic system. Thus, our third objective was to establish whether there was a loss and/or decreased somal/nuclear volume of midbrain dopaminergic neurons in the same animal model. Some of our behavioural and anatomical (stereological) analyses were completed in the same 16–18-month-old animals. Hence, we also examined whether any correlations existed between behaviour and anatomy as our fourth objective.

The context for this research is as follows: ADHD is the most prominent psychiatric condition in children, affecting ~5.3%, and persists into adulthood for ~2.5% of the population [1]. ADHD is a debilitating disorder, with a high societal cost exceeding USD 250 billion per year in the USA [2,3]. Existing pharmacotherapy for ADHD is not designed for the diversity of symptom presentations and therefore lacks overall efficacy [4]. It also has a large side-effect burden [4]. For improved pharmacotherapy and for prevention, carefully validated animal models are required to enhance our understanding of each presentation/subtype [4].

The DSM-V (American Psychiatric Association, 2013, [5]) identifies three presentations of ADHD: (i) predominantly ADHD inattentive (ADHD-I), (ii) predominantly ADHD hyperactive and impulsive (ADHD-HI), and (iii) ADHD hyperactive, impulsive, and inattentive (combined, ADHD-C) presentation [4]. Previously, in the DSM-IV, these presentations were identified as subtypes (American Psychiatric Association, 2000 [6]). The change to presentations emphasized that symptom clusters can change as patients mature [1]. In humans, attention problems are typically described as distractibility and trouble with sustaining attention [7]. Hyperactivity is typically seen in restlessness and fidgeting (i.e., inability to sit still) [7]. Impulsiveness is reflected in an inability to withhold inappropriate responses, such as premature responding, overly rapid responsiveness, excessive attraction to immediate reward, acting without reflecting, recklessness, and impetuous behaviour (i.e., may interrupt conversations) [7]. In humans with ADHD and in animal models, a specific behavioural testing paradigm can be used to detect hyperactivity and impulsivity in response *to delayed reward*, a *hallmark* of ADHD, and inattention [7,8].

There are reports of animal models for the ADHD-I presentation for rats [9,10], the ADHD-C presentation for rats and mice [8,11,12,13], and the ADHD-HI presentation for mice [14,15,16,17]. However, there is no published rat or mouse model of the ADHD-HI presentation that specifically tested ADHD-like hyperactivity/impulsivity in response to delayed reward, a hallmark of ADHD (see also below and Section 3). This specific test was undertaken in rats in the current study.

A key characteristic of the animal models is that they generally mimic genetic predispositions or environmental factors that increase susceptibility to ADHD [18]. The genetic models include the most extensively studied spontaneously hyperactive rats [8,19], as well as Wistar/Kyoto rats from Charles River [10]. The genetic models also include mice with a knockout of either the dopamine transporter [14] or the neurokinin 1 receptor [15], or with overexpression of Ataxin-7 [17]. Environmental factors thought to contribute to ADHD have been modelled by, for example, prenatal nicotine exposure in mice [12], postnatal exposure of rats to polychlorinated biphenyl congeners (i.e., industrial chemicals) [11], or hypoxic exposure of young rats [20,21]. In some rat models, normal animals have been behaviourally characterized and a subgroup of the animals that exhibited ADHD-like phenotypes were compared with the other animals (e.g., [9,13,16]). This array of animal models may potentially indicate multiple causes of ADHD. In the current study, an important environmental factor that increases susceptibility to ADHD was investigated—specifically, the effect of repeated hypoxia during the equivalent of extreme prematurity in rats (see further details below).

Diagnosis of ADHD presentations/subtypes is behaviourally based. Hence, the validation of an animal model should be based first on behaviour [10]. Specifically, evidence of the presence or absence of ADHD-like hyperactivity, ADHD-like impulsivity, and ADHD-like inattention is required. In animal models of ADHD, the open field test has been used extensively to measure hyperactivity [18]. Yet, the open field test has poor reliability as a measure of ADHD-like hyperactivity (Wickens et al. [22], see also Section 3). Instead, hyperactivity in response to delayed reward should be primarily investigated, since it is a characteristic feature of ADHD [7]. It is rarely investigated, however. It has been investigated in spontaneously hypertensive rats, which showed hyperactivity in response to delayed reward as part of the evidence for the ADHD-C presentation [8].

We developed an animal model in which male Sprague-Dawley rats were ADHD-like hyperactive in response to delayed reward, with no attention deficit, when tested from 16 months of age [20]. These rats were exposed to repeated hypoxic brain injury during the equivalent of extreme prematurity [20,21]. Extremely premature birth (22–28 weeks of gestation) is strongly associated with the development of ADHD [23,24,25]. Males are more vulnerable [26]. An investigation of ADHD-like impulsivity in response to delayed reward [8] was not undertaken in Oorschot et al. [20] due to time constraints. Here, using recorded data from the same cohort in Oorschot et al. [20], we first investigated whether male Sprague-Dawley rats exposed to repeated hypoxic brain injury during the equivalent of extreme prematurity were ADHD-like impulsive from 16 months of age. Since impulsivity or inattention can be outgrown during maturity, ADHD-like impulsivity and hyperactivity in response to delayed reward [8] and inattention were also investigated in a different male cohort at 3 months of age.

In humans, the hyperactive/impulsive presentation/subtype is correlated with decreased activation of the ventral striatum in adult males, and in adolescents, with ADHD [27,28]. Since dopaminergic neurons in the midbrain VTA primarily innervate the ventral striatum, the death of these neurons may contribute to the ADHD-like hyperactivity observed in repeated hypoxia rats at 16 months of age [20]. If this occurs, future novel pharmacotherapies could target their rescue early, at birth, to facilitate a permanent cure. This is in marked contrast to current drugs that provide only temporary symptomatic relief from 3–5 years of age [1]. It is unknown, however, whether the death or a reduced somal volume of midbrain dopaminergic neurons contribute to ADHD-like hyperactivity in repeated hypoxia male rats. Thus, we stereologically measured the absolute number and the average somal/nuclear volume of immunostained dopaminergic neurons in the midbrain of 18-month-old rats and in a third cohort of rats at 2 weeks of age. The right midbrain was analysed because dopaminergic deficits are evident in the right cerebrum in human ADHD (e.g., [29,30]) and dopaminergic neurons in the right midbrain innervate neurons in the right cerebrum [31]. Specifically, dopaminergic neurons located in the VTA and three other midbrain regions for comparison [32] were analysed. Behavioural versus anatomical correlations were also completed because ADHD-like hyperactivity and ADHD-like impulsivity at 16 months of age and the absolute number of dopaminergic neurons at 18 months of age were measured in the same animals. This strategy contributes to the lead taken by preclinical animal research in increasing the understanding of a potential pathological mechanism of a neurodevelopmental disorder such as ADHD. The aim of this type of research is to enhance the search for novel targets and new neuroprotective strategies [33].

It is acknowledged that increasing evidence also suggests abnormalities in networks within the prefrontal cortex, or frontal–striatal dysfunction, in ADHD [34,35,36,37]. Catecholamine dysregulation is thought to be the underlying pathophysiological substrate [34,35,36,37]. Since the dopaminergic neurons of the VTA also innervate the prefrontal cortex [38], they may contribute to the abnormalities in these networks. The prefrontal cortex also has a crucial role in cognitive flexibility, which is the ability to adapt behaviour in response to changes in the environment [39]. Cognitive flexibility is impaired in ADHD [40]. Potential pathological mechanisms generating this impairment are deficits in dopaminergic signalling [39] and altered tryptophan–kynurenine metabolism [33]. Due to the extensive behavioural and stereological experiments outlined herein, investigation of networks in the prefrontal cortex and cognitive flexibility was beyond the scope of the current study. They should be investigated in future studies (see also Section 3).

## 2. Results

### 2.1. Repeated Hypoxia Did Not Induce ADHD-like Impulsivity at 16 Months of Age

**Figure 1:**
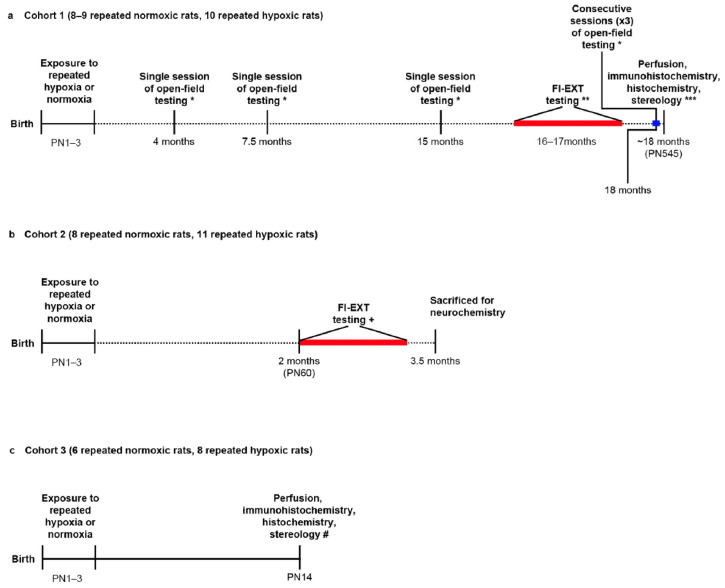
Timelines for the experiments. (**a**) Experimental timeline for the long-term cohort of repeated normoxia and repeated hypoxia rats (Cohort 1). This cohort was used for open field testing at 4, 7.5, 15, and 18 months of age, fixed interval–extinction (FI-EXT) testing from 16 months of age, and histochemical and immunohistochemical investigation of midbrain neurons. * Data from testing on the open field are shown in this paper. ** Hyperactivity and inattention data were published in Oorschot et al. [20]. Impulsivity data are shown in this paper. *** Myelin data were published in Oorschot et al. [21]. Data on the absolute number of midbrain dopaminergic neurons and total absolute number of thionin-stained neurons are shown in this paper. The nuclear volume data are shown in this paper. (**b**) Experimental timeline for the short-term cohort of repeated normoxia and repeated hypoxia rats (Cohort 2). This cohort was used for FI-EXT testing from 2 months of age. The animals were sacrificed at 3.5 months of age for neurochemical experiments and neurochemical/behavioural correlations that are not reported in this paper. (**c**) Experimental timeline for the short-term cohort of repeated normoxia and repeated hypoxia rats (Cohort 3). These rats were perfused at PN14 for histochemical, immunohistochemical, and stereological investigation of midbrain neurons. # Data are shown in this paper. Abbreviation: PN, postnatal day.

Rats were tested on a fixed-interval (FI)–extinction (EXT) testing schedule of reinforcement in operant chambers from 16 months of age (cohort 1; Oorschot et al. [20]) (Figure 1a) or from PN60 (cohort 2) (Figure 1b). Each FI period was used to test whether an animal was impulsive and/or hyperactive in response to delayed reward [8]. Impulsivity was measured by analysing inter-response times (IRTs) between lever presses during the FI task. A large number of left lever presses with short IRTs (<1.00 s) indicated impulsive responding with short bursts of activity [41,42].

**Figure 2:**
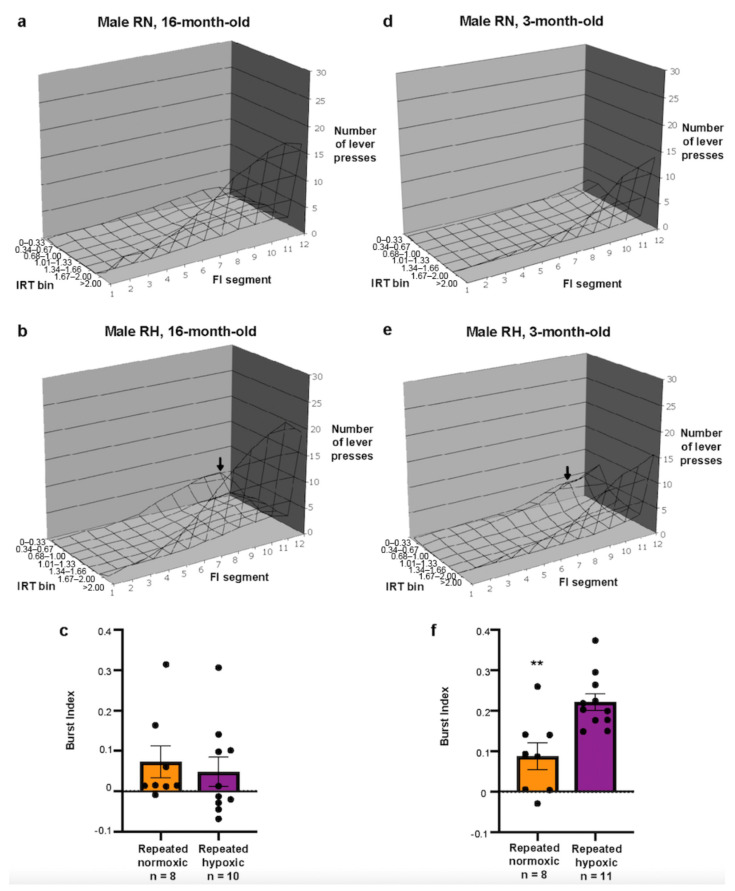
Measurement of impulsivity in male 16-month-old and 3-month-old rats. (**a**,**b**) Impulsivity data showing the incidence of different inter-response times (IRTs) between lever presses during each fixed interval (FI) task of two min for (**a**) repeated normoxia (RN) rats and (**b**) repeated hypoxia (RH) rats. These rats were tested from 16 months of age. IRTs were distributed into inter-response (IRT) bins of 0.33 s duration, spanning from 0 to >2 s. Each FI period of two min (120 s) was divided into 12 FI segments, each with a duration of 10 s. FI segment 1 is the first 10 s in each time period of 120 s, FI segment 2 is the second 10 s in each time period of 120 s, and so on. (**a**) versus (**b**), repeated measures ANOVA, *p* < 0.292. The arrow in (**b**) indicates a higher number of IRTs of 0–0.33 s duration compared to (**a**). (**c**) Burst index data for 16-month-old repeated normoxia and repeated hypoxia rats, two-tailed Student’s *t*-test, *p* = 0.654. (**d**,**e**) Data for impulsivity during FI testing for 3-month-old repeated normoxia (RN) rats (**d**) and repeated hypoxia (RH) rats (**e**), (**d**) versus (**e**), repeated measures ANOVA, *p* < 0.0001. The arrow in (**e**) indicates a significantly higher number of IRTs of 0–0.33 s duration compared to (**d**). (**f**) Burst index data for 3-month-old repeated normoxia and repeated hypoxia rats, two-tailed Student’s *t*-test, ** *p* < 0.01.

The repeated normoxia and the repeated hypoxia groups had similar patterns of inter-response time (IRT) distributions across each bin of FI, with a high number of long IRTs (i.e., >2.00 s bin) over the last 60 s of the FI segment (Figure 2a,b). The repeated hypoxia rats appeared to have a greater number of short IRTs (i.e., in bin 0–0.33 s, see arrow in Figure 2b) than the repeated normoxia rats over the last 60 s of FI, although there was no significant difference in the IRT distribution between the groups (repeated measures ANOVA, F_(1,16)_ = 1.185, *p* < 0.292, Figure 2a,b).

IRT distributions across FI may be influenced by a high overall lever press response rate [42]. To measure impulsivity independently of response rate, the burst index was calculated. The repeated hypoxia group did not have a significantly higher burst index (0.0487 ± 0.0382, mean ± SEM) than the repeated normoxia group (0.0732 ± 0.0421, t_16_ = 0.4566, *p* = 0.654, two-tailed Student’s *t*-test, Figure 2c).

### 2.2. Repeated Hypoxia Induced ADHD-like Impulsivity at 3 Months of Age 

The repeated hypoxia rats had a statistically significant increase in the number of short IRTs (i.e., bins < 1.00 s, see arrow in Figure 2e) over the last 60 s of FI than the repeated normoxia rats (repeated measures ANOVA, F_(1,17)_ = 52, *p* < 0.0001, Figure 2d,e). The average burst index for the repeated hypoxia rats (0.22 ± 0.02, mean ± SEM) was significantly higher than that of the repeated normoxia rats (0.09 ± 0.04, t_17_ = 3.595, *p* < 0.01, two-tailed Student’s *t*-test, Figure 2f). These IRT distributions and burst index values strongly indicate [41,43] that young repeated hypoxia rats responded impulsively with an excess of short bursts of activity.

### 2.3. Repeated Hypoxia Induced ADHD-like Hyperactivity at 3 Months of Age

**Figure 3:**
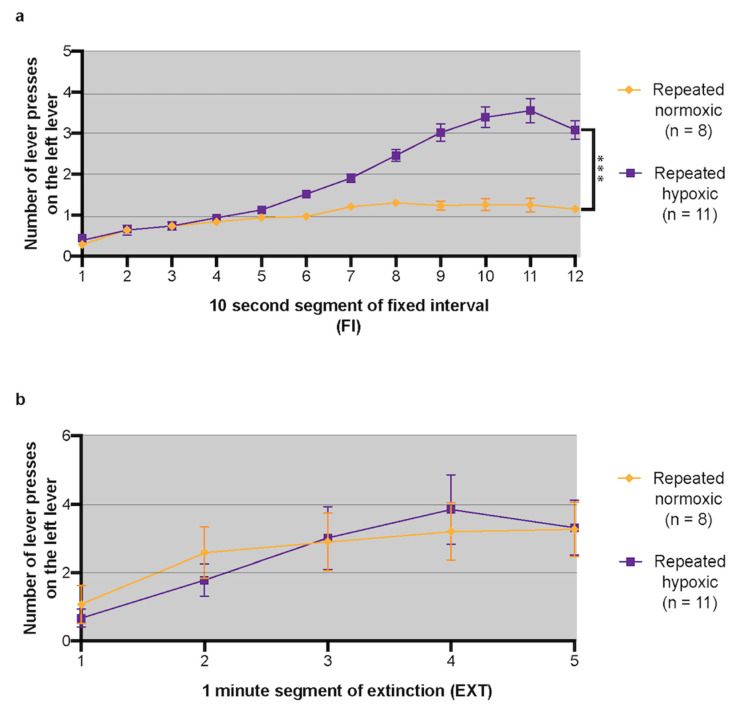
Behavioural response during the fixed interval (FI) and extinction (EXT) component of a multiple FI-EXT schedule in repeated normoxia and repeated hypoxia rats at 3 months of age. (**a**) Average number of lever presses made during each bin of FI for days 23–32 for repeated normoxia and repeated hypoxia rats, repeated measures ANOVA, *** *p* < 0.0001. (**b**) Average number of lever presses made during each bin of EXT for days 23–32.

For the FI task, rats had to press the left lever to receive a food reward after a delay of 2 min. Over the course of the 2 min bins of FI, a characteristic scalloped curve develops whereby lever press responding increases over time as the delay increases [42]. This is known as the delay-of-reinforcement gradient. Humans with ADHD and animal models of ADHD such as the spontaneously hypertensive rat have a steeper and shorter delay-of-reinforcement gradient than normal, representing a hyperactive response to delayed reward (i.e., there is an intolerance to delayed reinforcement [42]). Similar to the 16-month-old repeated hypoxia and repeated normoxia male rats tested previously [20], the pattern of responding during FI for the PN60 rats began to stabilise into the typical scalloped-shaped curve around day 7, although response rates generally continued to be unstable until day 20 of testing. Stable performance for a group was defined as when the average number of presses on the left lever was <0.5 for the 1st 10 s bin of each FI and was > 1.5 for the 11th 10 s bin of each FI. During days 23–32 of testing on FI, when the rats were ~3 months of age, the repeated hypoxia rats had an increased terminal lever press rate and made significantly more left lever presses over the last 60 s of each 120 s FI period than the repeated normoxia rats (i.e., there was an interaction between time and treatment, repeated measures ANOVA, F_(1,17)_ = 30.14, *p* < 0.0001, Figure 3a). When the treatments were compared (i.e., a between-subjects effect) there was also a significant difference between the repeated hypoxia and repeated normoxia rats (repeated measures ANOVA, F_(1,17)_ = 39, *p* < 0.0001, Figure 3a). Thus, young adult repeated hypoxia rats were hyperactive in response to delayed reward, with the same effect detected at 16 months of age by Oorschot et al. [20].

### 2.4. Repeated Hypoxia Did Not Induce an ADHD-like Attention Deficit at 3 Months of Age

The EXT component of the FI-EXT task measures sustained attention and sensitivity to change [41,43]. Sensitivity to change is measured by the switch from the lights-on FI periods to the lights-off EXT period where no reward is available. Both ADHD subjects and controls stop responding for a period of time after the beginning of EXT, which indicates a normal sensitivity to a change in stimulus [42]. However, after a short period of time, ADHD-like subjects with deficits in sustained attention resumed responding at higher levels than controls despite not being rewarded during this EXT period [42]. Responses during the first 20 days of EXT for both groups of rats were generally unstable. By day 23, responses had stabilised and remained stable throughout the last 10 days of testing. Both the repeated hypoxia and repeated normoxia groups had a normal sensitivity to the change in stimulus from FI to EXT, as indicated by the cessation of pressing at the beginning of EXT (Figure 3b). There were no significant differences between the repeated hypoxia and repeated normoxia rats in the number of lever presses made during EXT (repeated measures ANOVA, F_(1,17)_ = 0.89, *p* < 0.36, Figure 3b) or in the pattern of responding. Thus, repeated hypoxia young adult male rats had no attention deficit, with the same effect being detected at 16 months of age by Oorschot et al. [20].

### 2.5. Repeated Hypoxia Rats Were Not Hyperactive in the Open Field Test

**Figure 4:**
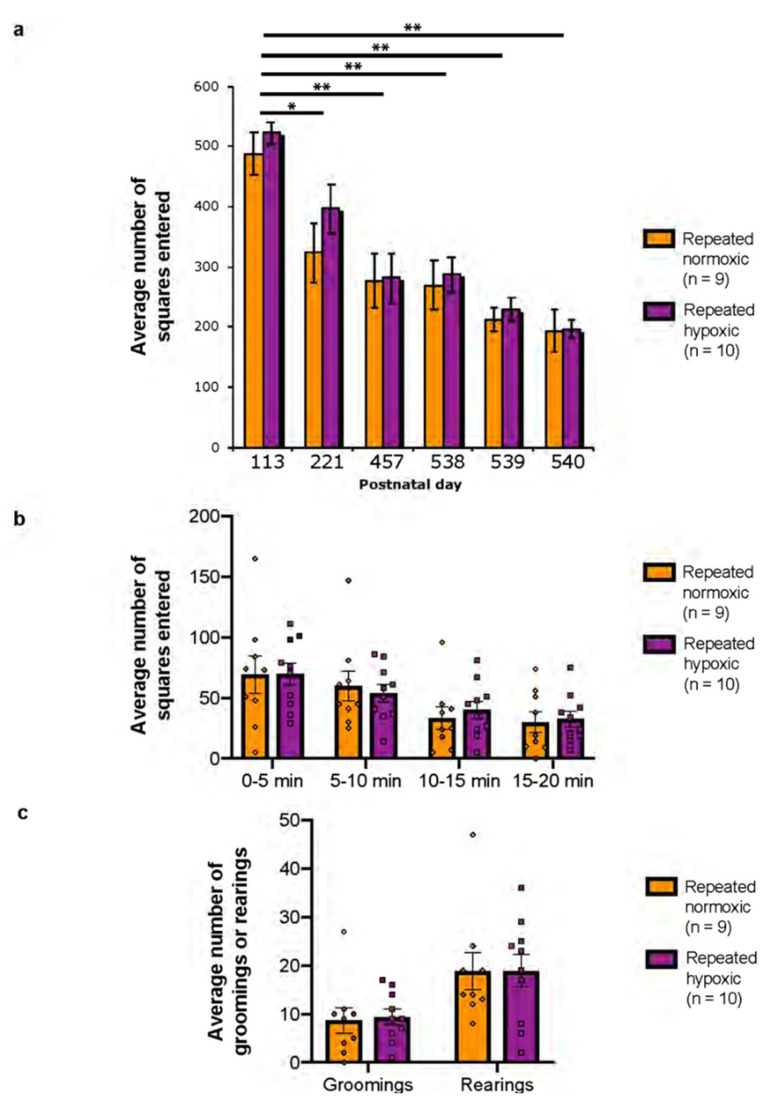
Open field behaviour in repeated normoxia and repeated hypoxia rats at different ages. (**a**) Average number of squares entered in each session of the open field test at postnatal day (PN) 113, 221, 457, and 538–540, * *p* < 0.05 versus PN113 for both groups, ** *p* < 0.01 versus PN113 for both groups, Bonferroni post-hoc analysis corrected for multiple comparisons. (**b**) Average number of squares entered within each 5 min period of a 20 min session of the open field test at PN540. (**c**) Average number of groomings or rearings in a 20 min session at PN540. Each circle is the number of squares entered (**b**) or groomings or rearings (**c**) achieved by an individual repeated normoxia rat and each square is the number achieved by an individual repeated hypoxia rat.

**Figure 5:**
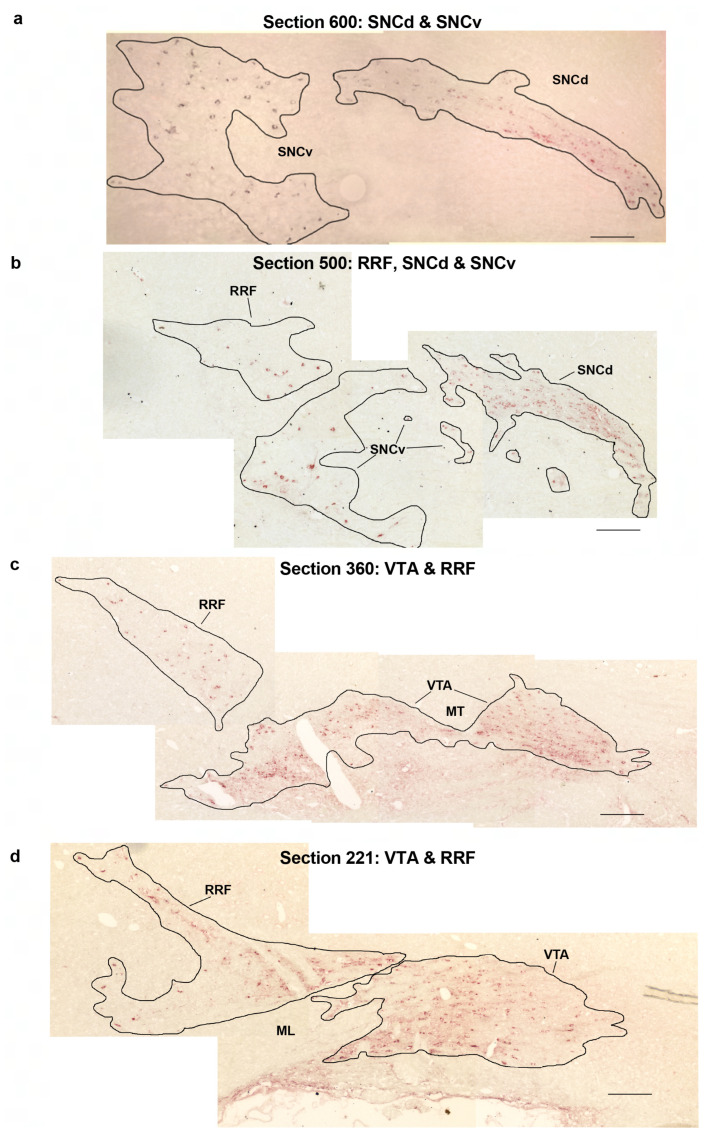
Anatomical regions investigated. Light microscopic images of sagittal sections of the rat midbrain showing the boundaries used to define the subregions (see also Section 4 for further details). Each subregion contained dopaminergic neurons immunostained with tyrosine hydroxylase. (**a**) Substantia nigra compacta dorsal tier (SNCd) and substantia nigra compacta ventral tier (SNCv); (**b**) retrorubral field (RRF), SNCd and SNCv, and (**c**,**d**) RRF and ventral tegmental area (VTA). Note that the left side of each image is posterior and the right side of each image is anterior in the brain. White matter tracts assist in the demarcation of boundaries, specifically the medial terminal nucleus (MT) in (**c**) and the medial lemniscus (ML) in (**d**). Note that these images are sampled from more than one rat. Scale bar: (**a**,**c**,**d**) 140 µm; (**b**)160 µm.

The same rats that were tested on the FI-EXT task at 16 months of age were tested in the open field at several younger ages (Figure 1a). The open field test was used as another measure of hyperactivity. Repeated hypoxia rats generally entered more squares per session than the repeated normoxia rats, although this was not significant at any of the ages tested (Figure 4a). In the three consecutive daily sessions at 18 months of age there were no significant differences between the groups on any of the days. This suggests that the repeated hypoxia rats did not display motor hyperactivity in the open field task. The total number of squares traversed in a 20 min session for both groups was significantly decreased between the two sessions at PN113 and PN221 compared with the sessions at PN457, 538, 539, and 540 (repeated measures ANOVA, F_(5,85)_ = 27.10, *p* < 0.0001; Figure 4a). In addition, both groups entered significantly less squares in the session at PN221 compared to that at PN113 (*p* < 0.05). When each 5 min period of a 20 min session was analysed, there was also no significant difference in the number of squares entered between the groups for all ages analysed (e.g., at PN540, Figure 4b). In a 20 min session, the total number of groomings and the total number of rearings did not differ significantly between the groups for all ages analysed (e.g., at PN540, Figure 5c). These data suggest that simple tests of locomotor activity, undertaken during the light phase of the day, may not accurately detect the type of hyperactivity seen in ADHD, particularly in aged rats. There was also a strong effect of age on performance in this task in male Sprague-Dawley rats, even in early adulthood.

### 2.6. Repeated Hypoxia Rats at PN14 and at 18 Months of Age Have a Decreased Absolute Number of Dopaminergic Neurons in the Right VTA but Not the Right Substantia Nigra Compacta or Retrorubral Field 

As indicated in the introduction, it is possible that the ADHD-like hyperactivity observed in repeated hypoxia rats at 16 months of age [20] and at 3 months of age (Figure 3) could be due to deficits in the midbrain dopaminergic system. It is unknown, however, whether the death of midbrain dopaminergic neurons could be a contributing factor to the development of ADHD-like hyperactivity. Thus, we stereologically measured the total (i.e., absolute) number of immunostained dopaminergic neurons in the right midbrain of 18-month-old rats (i.e., the cohort 1 rats) and in a new third cohort of PN14 repeated normoxia versus repeated hypoxia rats (Figure 1c). The PN14 cohort consisted of repeated hypoxia and repeated normoxia rats from two different litters.

The rationale for specifically analysing the right midbrain for both cohorts is indicated in Section 1. The rationale for completing anatomical experiments at PN14 on the right midbrain dopaminergic neurons is as follows: Using perfusion-fixed rats at PN14, we assessed short-term anatomical changes in myelin [21] because a deficit in cerebral myelin is a hallmark of extreme prematurity. Myelin is also present in the rat cerebrum by PN14. We analysed myelin in the *left* cerebral hemisphere [21]. Here, we analysed the *right* cerebral hemisphere of the same PN14 rats for potential effects on the dopaminergic system. This is because these effects are hypothesised in the literature to be right-sided and should be evident by this age, if present, after repeated hypoxia from PN1–3. This approach/rationale also used animals efficiently (see also Section 3). The sectioning, immunostaining, and stereology of the right cerebral hemispheres was completed immediately after the anatomical analyses of myelin in the left cerebral hemispheres.

Within the midbrain, major populations of dopaminergic neurons are located in the VTA, the caudal or central linear nucleus (CLi) of the VTA, the substantia nigra compacta dorsal (SNCd) and ventral (SNCv) tiers, and the retrorubral field (RRF) [32]. These specific populations (Figure 5a–d) were stereologically analysed.

**Figure 6:**
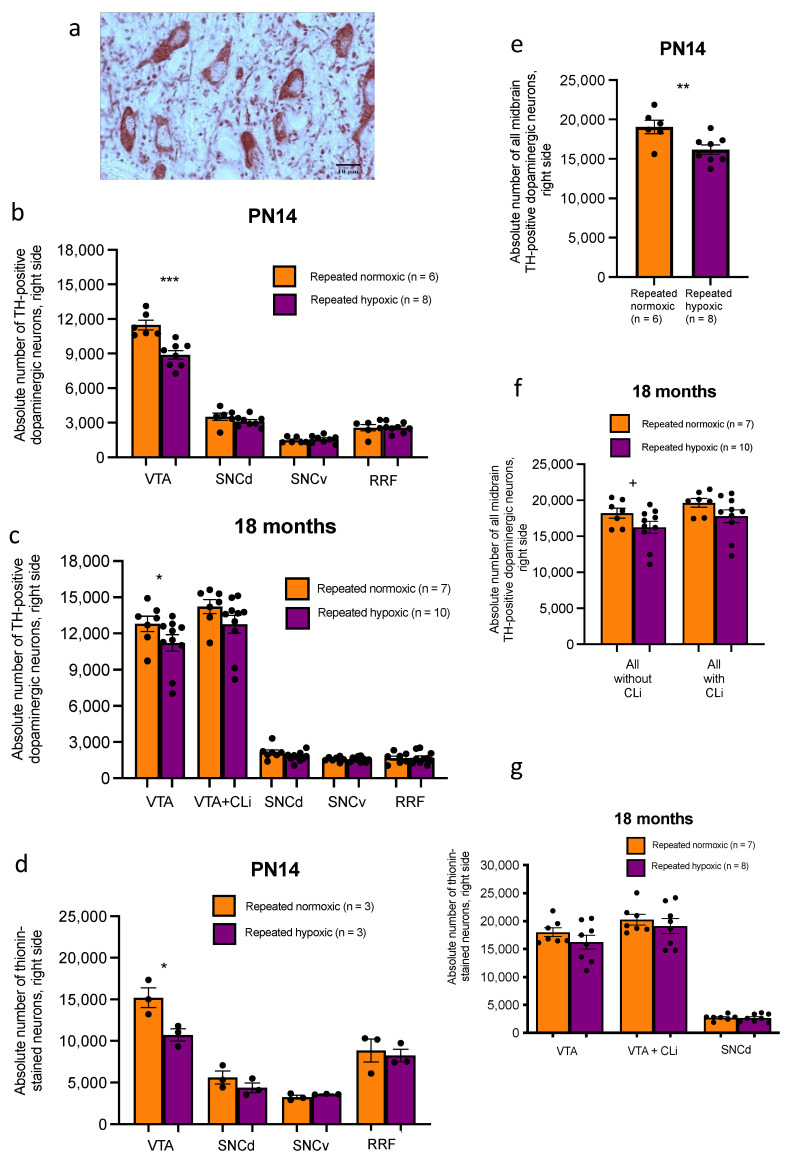
Absolute number of tyrosine hydroxylase (TH)-positive dopaminergic neurons and thionin-stained neurons in repeated normoxia and repeated hypoxia rats. (**a**) Immunostained TH-positive dopaminergic neurons in the VTA. Average absolute number of TH-positive dopaminergic neurons in each midbrain dopaminergic subregion at PN14 (**b**) and at 18 months of age (**c**). Average absolute number of thionin-stained neurons in each midbrain dopaminergic subregion at PN14 (**d**). Average combined (i.e., total) absolute number of TH-positive dopaminergic neurons in the midbrain at PN14 (**e**) and at 18 months of age (**f**). (**g**) Absolute number of thionin-stained neurons in the ventral tegmental area (VTA), VTA plus caudal linear nucleus (CLi), and the substantia nigra compacta dorsal tier (SNCd), at 18 months of age. * *p* ≤ 0.05, + *p* = 0.0561; ** *p* < 0.02, *** *p* < 0.001. RRF, retrorubral field; SNCv, substantia nigra compacta ventral tier.

Repeated hypoxia rats at PN14 had a significant decrease in the mean absolute number of dopaminergic (i.e., tyrosine hydroxylase (TH)-positive, Figure 6a) neurons in the right VTA (8894 ± 400) compared to repeated normoxia rats (11,479 ± 466, two-tailed Student’s *t*-test, t12 = 4.548, *p* = 0.0007, Figure 6b). There were no significant differences in mean absolute neuronal number in the SNCd (3119 ± 175 versus 3515 ± 346), the SNCv (1586 ± 122 versus 1489 ± 112), or the RRF (2567 ± 167 versus 2578 ± 303; all *p* > 0.05; SNCd, t12 = 1.198; SNCv, U = 20; RRF, t12 = 0.036; Figure 6b). There was also a significant decrease in the mean absolute number of all dopaminergic neurons in the right midbrain (16,166 ± 637) compared to repeated normoxia rats (19,060 ± 935, two-tailed Student’s *t*-test, t12 = 2.873, *p* = 0.0140, Figure 6e) at PN14. At 18 months of age there was still a significant decrease in the absolute number of dopaminergic neurons in the right VTA of repeated hypoxia rats (11,213 ± 708) compared to repeated normoxia rats (12,798 ± 687, one-tailed Mann–Whitney U test, U = 17, *p* = 0.0439, Figure 6c). There was also a trend of a decreased absolute number of dopaminergic neurons in the right VTA + CLi of repeated hypoxia rats (12,754 ± 775) compared to repeated normoxia rats (14,229 ± 629, one-tailed Mann–Whitney U test, U = 18, *p* = 0.0544). There were no significant differences in the SNCd (2151 ± 237 versus 1777 ± 131), the SNCv (1577 ± 79 versus 1567 ± 70), or the RRF (1674 ± 181 versus 1691 ± 166; SNCd, U = 17, *p* = 0.088; SNCv, t15 = 0.098; RRF, t15 = 0.073, all *p* > 0.05, two-tailed tests; Figure 6c). There was also a trend of a decreased absolute number of dopaminergic neurons in the entire right midbrain, excluding the CLi, of repeated hypoxia rats (16,247 ± 878) compared to repeated normoxia rats (18,200 ± 749, one-tailed Student’s *t*-test, t15 = 1.688, *p* = 0.0561, Figure 6f). There was no significant decrease in the mean absolute number of all dopaminergic neurons in the right midbrain (17,789 ± 943) compared to repeated normoxia rats (19,630 ± 650, one-tailed Student’s *t*-test, t15 = 1.549, *p* = 0.0711, Figure 6f) at 18 months of age. In summary, for the right midbrain, there was only a loss of dopaminergic neurons specifically in the VTA in the repeated hypoxia rats. This was a long-term effect up to 18 months of age.

More detailed data on the total reference volume of the VTA and the numerical density of the dopaminergic neurons in the VTA are provided in Table 1 for both experimental conditions at PN14 and PN545. From these data it is evident that there was a 10–16% decrease in numerical density for the repeated hypoxia versus the repeated normoxia rats at both ages and that this contributed to the outcome of the absolute number. There was also a 14% decrease in the total reference volume at PN14, and this also contributed to the outcome of the absolute number. Hence, these findings show the elements that contributed to the observed decrease in the absolute number of dopaminergic neurons in the VTA for the repeated hypoxia group versus the repeated normoxia group.

Of note, the absolute number of dopaminergic neurons in the VTA was the only statistically significant outcome at both ages. The numerical density data and the total reference volume data were both not statistically significant (Table 1). These results confirm and extend previous results in the field of stereology that conclusions based on numerical density alone or on total reference volume alone should be avoided since they are indirect measures of neuronal survival—both measures can lead to misinterpretations of biological processes [44,45,46,47,48,49]. It is the absolute number of surviving neurons that generally leads to more reliable conclusions.

To investigate whether any loss of TH-positive neurons was due to actual neuronal loss or a loss of TH protein expression, the sections from a subset of the same brains were stained with thionin. Thionin is a nuclear stain that stains all neurons. Repeated hypoxia rats at PN14 had a statistically significant decrease in the absolute number of thionin-stained neurons in the right VTA (10,723 ± 901) compared to repeated normoxia rats (15,193 ± 1458, two-tailed Student’s *t*-test, t_4_ = 3.194, *p* = 0.0331, Figure 6d). This decrease of 29% was very similar to the decrease of 23% reported for dopaminergic neurons in the right VTA. Thus, the decrease in the right VTA is most likely to have been due to neuronal loss rather than a loss of expression of the tyrosine hydroxylase protein.

There was no difference in the absolute number of thionin-stained neurons at PN14 in the right SNCd, right SNCv, or right RRF for the repeated hypoxia versus repeated normoxia rats (SNCd, 4369 ± 718 versus 5611 ± 956, t_4_ = 1.273; SNCv, 3600 ± 29 versus 3250 ± 281, t_4_ = 1.518; RRF, 8237 ± 925 versus 8861 ± 1694, *U* = 3; all *p* > 0.20, Figure 6d). Thus, for the right midbrain, neuronal loss only occurred in the VTA in the repeated hypoxia rats.

In contrast to the PN14 rats, there was no significant difference in thionin neuronal number in the VTA + CLi of repeated hypoxic rats at 18 months of age (19,117 ± 1408) compared to repeated normoxia rats (20,268 ± 1021, one-tailed Student’s *t*-test, t_13_ = 0.6912, *p* < 0.25, Figure 6g). There was also no significant difference in thionin neuronal number for the VTA without the CLi at 18 months of age (16,230 ± 1314) compared to repeated normoxia rats (18,015 ± 869, one-tailed Student’s *t*-test, t_13_ = 1.176, *p* < 0.13). Hence, for a subgroup of rats at 18 months of age, a general thionin stain did not detect a general neuronal loss in the VTA. This may have been due to the smaller n per group used in this analysis and an age-related effect.

### 2.7. Repeated Hypoxia Had No Effect on Midbrain Dopaminergic Somal/Nuclear Volume

A reduced somal or nuclear volume, potentially reflecting decreased metabolic activity, of dopaminergic neurons in the right midbrain may have contributed to ADHD-like hyperactivity in repeated hypoxia male rats. Hence, the somal volume of SNCd and VTA dopaminergic neurons was measured in the same 5 µm sections that were used to stereologically measure the absolute number of TH-positive neurons in the right midbrain at PN14 (i.e., in the 8 repeated hypoxia and 6 repeated normoxia male rats; Figure 1c). The nuclear volume of the dopaminergic neurons in the VTA was measured at PN545.

**Figure 7:**
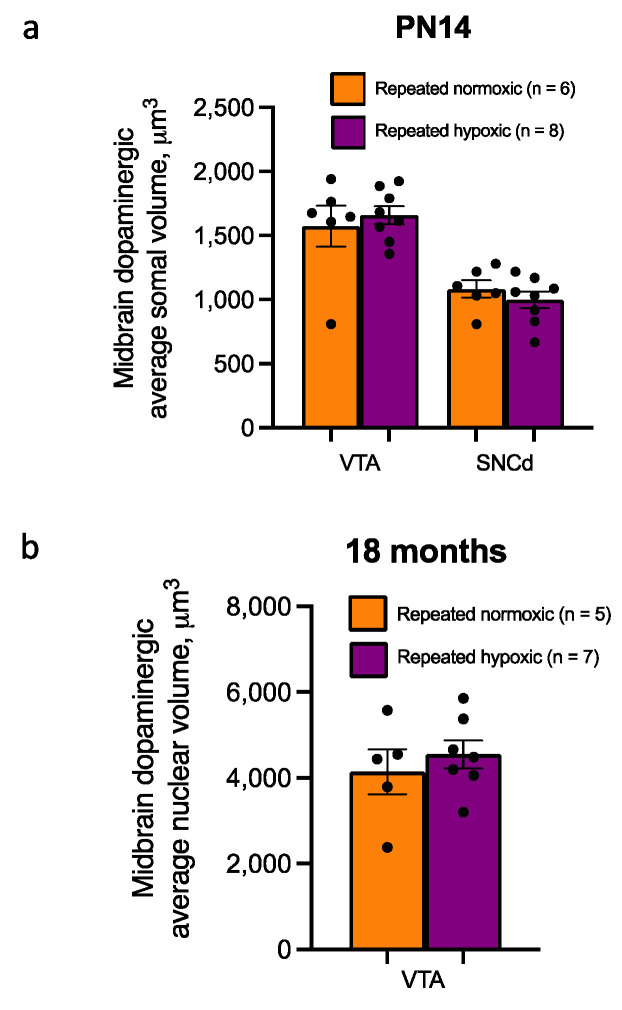
Somal/nuclear volume of tyrosine hydroxylase (TH)-positive dopaminergic neurons in repeated normoxia and repeated hypoxia rats. Average somal/nuclear volume of TH-positive dopaminergic neurons in (**a**) the ventral tegmental area (VTA) and substantia nigra compacta dorsal tier (SNCd) at PN14, and in the VTA (**b**) at 18 months of age.

For the dopaminergic neurons in the VTA and SNCd, there was no significant difference in the average somal volume for the repeated hypoxia versus repeated normoxia rats at PN14 (VTA: repeated hypoxia, 1659 ± 76 µm^3^, repeated normoxia, 1574 ± 176 µm^3^, two-tailed Mann–Whitney *U* test, *U* = 24, *p* > 0.9999; SNCd: repeated hypoxia, 998 ± 69 µm^3^, repeated normoxia, 1083 ± 74 µm^3^, two-tailed Student’s *t*-test, t_12_ = 0.900, *p* = 0.3859, Figure 7a). At 18 months of age, there was no significant difference in the nuclear volume of dopaminergic neurons in the VTA (repeated hypoxia, 4547 ± 357 µm^3^, n = 7; repeated normoxia, 4145 ± 589 µm^3^, n = 5, two-tailed Student’s *t*-test, t_10_ = 0.682, *p* = 0.5108, Figure 7b).

### 2.8. Reliable Stereological Data

For the stereological estimates, estimates of precision (i.e., the coefficient of error (CE)) were calculated. The mean CEs for the Vref, N_V_, *N*, and somal/nuclear volume measurements predominantly ranged from 1 to 10% (Table 2 and Table 3; 97 of 102 group measurements). A mean CE of 10% or less is generally considered to yield a reliable estimate because the variation (or CV = SD/mean) between animals for the parameter being assessed is usually at least 10–15% [50]. A minority of the mean CEs ranged between 11 and 13% (5 of 102, Table 2 and Table 3). The observed mean variance of the Vref, N_V_, *N*, and somal/nuclear volume (i.e., CE^2^) was less than half of the total variance for each parameter for the respective group (i.e., CV^2^, Table 3 and Table 4) for 95 of 102 measurements. This indicates that most of the variation was due to biological variation rather than variation in the precision of the estimates made with the stereological techniques employed [51,52,53]. Reliable estimates were thus obtained for these measurements [51,52,53]. When the CE^2^/CV^2^ ratio was not less than 0.5 (e.g., 1.43 [(0.122)^2^/(0.102)^2^] for the N_V_ of dopaminergic neurons in the RRF of the repeated hypoxic group at PN545, Table 3; see also examples in Table 4), there was a low biological variance (i.e., CV, [50,52,54]) for the specific parameter (i.e., 0.2–10.2% for the N_V_ data, 1.2–13.4% for the *N* data, Table 3 and Table 4). A higher CE^2^/CV^2^ ratio is acceptable when the biological variance of a parameter is low [54].

For an explanation of the abbreviations used, see Section 4.

### 2.9. Changes in Midbrain Anatomy Correlate with Behaviour

**Figure 8:**
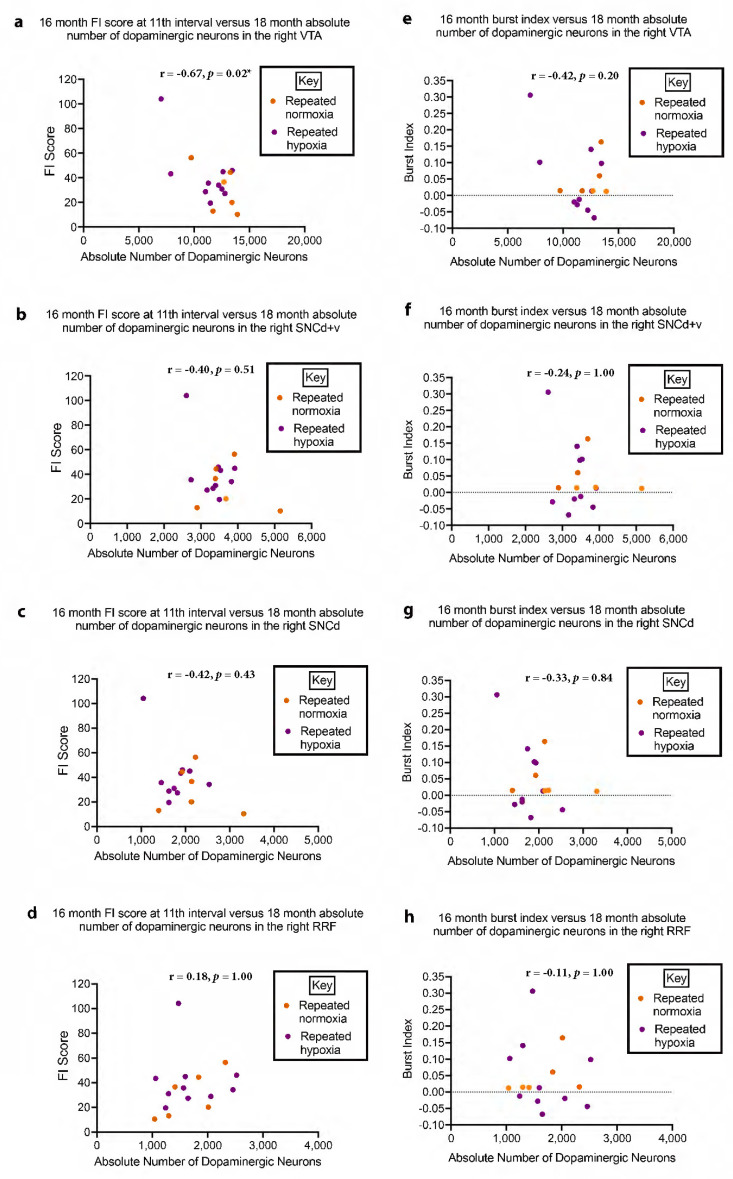
Relationship/correlation between the behavioural outcomes at 16 months of age and the absolute number of dopaminergic neurons at 18 months of age in repeated normoxia and repeated hypoxia rats. (**a**–**d**) Fixed interval (FI) at the 11th interval versus the absolute number of dopaminergic (TH-positive) neurons in the right ventral tegmental area (VTA) (**a**), right substantia nigra compacta dorsal tier (SNCd) combined (SNCd + v) with the right substantia nigra compacta ventral tier (SNCv) (**b**), right SNCd (**c**), and right retrorubral field (RRF) (**d**). (**e**–**h**) Burst index versus absolute number of dopaminergic (TH-positive) neurons in the right VTA (**e**), right SNCd + v (**f**), right SNCd (**g**), and right RRF (**h**). There is a statistically significant negative correlation for the VTA (**a**) but not for the SNCd + v, SNCd, or RRF (**b**–**d**,**f**–**h**). * *p* ≤ 0.05.

**Figure 9:**
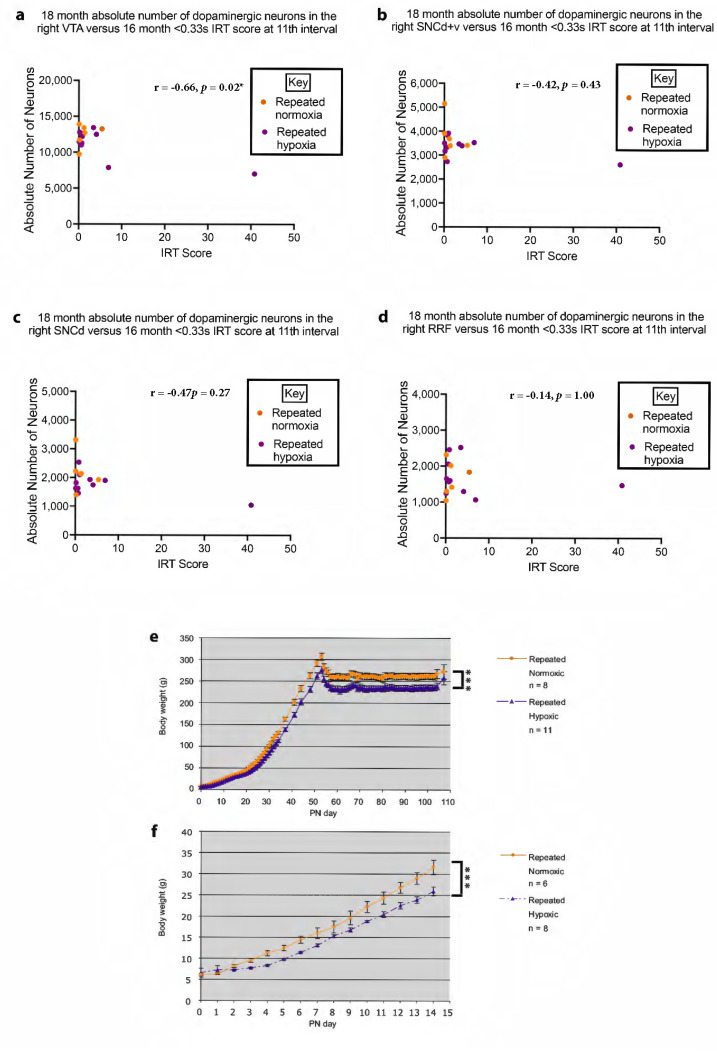
Relationship between the number of short inter-response times (IRTs) at 16 months of age and the absolute number of dopaminergic neurons at 18 months of age in repeated normoxia and repeated hypoxia rats, and body weight data for cohort 2 and cohort 3. (**a**) Absolute number of dopaminergic (TH-positive) neurons in the right ventral tegmental area (VTA) (**a**), right substantia nigra compacta dorsal tier (SNCd) combined (SNCd + v) with the right substantia nigra compacta ventral tier (SNCv) (**b**), right SNCd (**c**), and right retrorubral field (RRF) (**d**) versus the number of short (<0.33 s) IRTs at the 11th interval of the fixed interval component. There is a statistically significant negative correlation for the VTA (**a**), but not for the SNCd + v, SNCd or RRF (**b**–**d**). (**e**,**f**) Body weight data for cohort 2 (**d**) and cohort 3 (**e**) across their lifespan. (**e**,**f**) Both *p* < 0.001, indicating a long-term significant decrease in body weight in the repeated hypoxia rats. This was also observed for cohort 1. The body weight data for cohort 1 is published in Figure 5D in Oorschot et al. [21]. For (**a**–**d**), * *p* ≤ 0.05. For (**e**–**f**), please see Section 2 for the specific statistical details; *** *p* < 0.0001.

A statistically significant negative correlation/linear regression was observed for the absolute number of dopaminergic neurons in the right VTA at 18 months of age versus ADHD-like hyperactivity, and ADHD-like impulsivity with respect to IRT, at 16 months of age (Pearson’s r = −0.6737, *p* = 0.017, and Pearson’s r = −0.6574, *p* = 0.022, respectively, two-tailed, corrected for multiple comparisons, Figure 8a and Figure 9a). Hence, a lower value for the absolute number of dopaminergic neurons in the right VTA was correlated with a higher score for ADHD-like hyperactivity and impulsivity. A lower value for the absolute number of dopaminergic neurons in the right VTA did not correlate with the burst index measure of ADHD-like impulsivity (Pearson’s r = −0.4236, *p* = 0.200, Figure 8e). For the significant correlation with ADHD-like impulsivity, it is acknowledged that the rats did not show impulsivity based on the average burst index (Figure 2c). Yet, the impulsivity measure for the number of shorter IRTs was increased (see arrow in Figure 2b) for the pooled repeated hypoxic data (compare Figure 2a,b, although it is acknowledged this difference is not statistically significant). The significant difference became apparent when the data for inter-response times and the anatomical data for each individual rat were graphed and the association was statistically analysed (Figure 9a).

The absolute number of dopaminergic neurons in the right SNCd + v, right SNCd, and right RRF at 18 months of age did not correlate significantly with ADHD-like hyperactivity (SNCd + v, Pearson’s r = −0.3978, *p* = 0.5084; Figure 8b; SNCd, Pearson’s r = −0.4185, *p* = 0.4268; Figure 8c; RRF, Pearson’s r = 0.1810, *p* = 1.000, Figure 8d). In addition, the absolute number of dopaminergic neurons in the right SNCd + v, right SNCd, and right RRF at 18 months of age did not correlate significantly with ADHD-like impulsivity measured by the IRT (SNCd + v, Pearson’s r = −0.4182, *p* = 0.4280; Figure 9b; SNCd, Pearson’s r = −0.4697, *p* = 0.2656, Figure 9c; RRF, Pearson’s r = −0.1378, *p* = 1.000, Figure 9d) or ADHD-like impulsivity measured by the burst index (SNCd + v, Pearson’s r = −0.2448, *p* = 1.000, Figure 8f; SNCd, Pearson’s r = −0.3317, *p* = 0.8380, Figure 8g; RRF, Pearson’s r = −0.1055, *p* = 1.000, Figure 8h) at 16 months of age.

### 2.10. Repeated Hypoxia Rats Have a Significant Reduction in Body Weight

A significant long-term decrease in body weight was reported previously from PN2 onwards for the repeated hypoxia group versus the repeated normoxia group until 18 months of age (Oorschot et al. [20]; Figure 5D in Oorschot et al. [21]; cohort 1 in this paper). For cohort 2, a significant decrease in mean body weight was evident from PN0 to PN104 (the end of behavioural testing) in the repeated hypoxia group compared to the repeated normoxia group (F_1,17_ = 6.97, *p* < 0.0001, Figure 9e). There was also a significant effect of treatment (i.e., repeated hypoxic exposure (F_1,15_ = 14.76, *p* < 0.0001, Figure 9e). This significant difference commenced at PN3, the last day of hypoxic exposure (Bonferroni’s post-hoc test, t_13_ = 4.215, *p* < 0.002). For cohort 3, a significant decrease in mean body weight was evident from PN0 to PN14 in the repeated hypoxia group compared to the repeated normoxia group (F_1,14_ = 6.67, *p* < 0.0001, Figure 9f). There was also a significant effect of treatment (i.e., repeated hypoxic exposure (F_1,15_ = 14.76, *p* < 0.0001, Figure 9f). This significant difference commenced at PN3, the last day of hypoxic exposure (Bonferroni’s post-hoc test, t_13_ = 3.765, *p* < 0.003). Hence, the rats in cohorts 2 and 3 confirmed that a significant deficit in body weight commenced during repeated hypoxic exposures from PN1-3 and persisted thereafter.

## 3. Discussion

### 3.1. Major Findings

We demonstrated a novel animal model of the hyperactive/impulsive presentation of ADHD. The rat phenotype was induced in males exposed to repeated hypoxia over 3 days during the second-trimester equivalent of extreme prematurity (PN1-3; [20,21]). ADHD-like hyperactivity and ADHD-like impulsivity were evident in repeated hypoxia male rats in response to delayed reward, consistent with clinical findings [18,57]. Specifically, ADHD-like hyperactivity and ADHD-like impulsivity were evident in 3-month-old rats using a multiple component schedule of reinforcement that detects hyperactivity and impulsivity in response to delayed reward, as in ADHD children [7]. There was no ADHD-like inattentiveness in another test used to detect inattention in ADHD children. In the short and long term, repeated hypoxia rats had a specific significant loss of dopaminergic neurons in the right VTA, thereby providing new evidence of a site of pathology. ADHD-like hyperactivity and ADHD-like impulsivity both correlated with a decreased absolute number of dopaminergic neurons in the right VTA. This was not evident for the right SNC nor the RRF, thereby suggesting a specific anatomical/functional association. Future novel pharmacotherapies can now be used to target the rescue of right VTA dopaminergic neurons early, at birth. This could potentially lead to a permanent cure for the hyperactive/impulsive presentation of ADHD. If this is achieved, it would be in marked contrast to current drugs that provide only temporary symptomatic relief from 3–5 years of age [1].

In the short and long term, midbrain dopaminergic neurons in repeated hypoxia rats did not have a significant decrease in somal/nuclear volume compared to repeated normoxia rats. This suggests that there was no change in the metabolic activity of these neurons for the two groups of animals.

A major advantage of this novel rat model is its evidence of construct validity and face validity for ADHD. Increasing evidence indicates that extremely premature birth (22–28 weeks of gestation) is strongly associated with the development of ADHD [24]. Hence, this animal model has a disease aetiology similar to that of humans and thus has construct validity. Face validity requires the recapitulation of behavioural symptoms seen in human ADHD-HI. The presence of ADHD-like hyperactivity and ADHD-like impulsivity in response to delayed reward indicates that the animal model had this evidence. Other evidence supporting the model’s validity is that decreased cerebral white matter and grey matter volumes (reported earlier in Oorschot et al. [21]) are also observed in human ADHD [57].

In repeated hypoxia rats aged 4 to 18 months, hyperactivity was not detected in an open field test. This is consistent with clinical findings in children with ADHD. Specifically, the locomotor activity of children using grid-line crossings in a clinical playroom did not correlate significantly with ratings of hyperactivity by parents [58] or clinical diagnosis [59]. Therefore, the open field test has poor reliability as a measure of ADHD-like hyperactivity (see also Wickens et al. [22]). In addition, many different types of rodents are hyperactive in a novel environment like the open field test, but few are hyperactive in familiar environments [19]. ADHD-like hyperactivity after delayed reward was evident in repeated hypoxia rats in a familiar environment at 3 months of age (this study) and at 16 months of age [20]. This highlights our novel findings. These data at 3 months of age also confirm and extend the findings of Oorschot et al. [20] at 16 months of age.

Some mouse animal models of the ADHD-HI presentation have been reported [14,15,16,17], yet they did not measure ADHD-like hyperactivity in response to delayed reward. Therefore, our model is unique in unambiguously demonstrating a specific increase in ADHD-like hyperactivity in response to delayed reward. It is also unique due to the lack of confounding effects of an increase in general locomotor hyperactivity in the open field test. There is evidence for this confound in the widely used spontaneously hypertensive rat model of ADHD [60].

Correlation of ADHD-like hyperactivity and ADHD-like impulsivity with a decreased absolute number of dopaminergic neurons in the right VTA supports extensive previous findings implicating the mesolimbic and mesocortical systems in ADHD. For example, hypoactivity of the dopaminergic mesolimbic pathway between the VTA and ventral striatum is considered to play a role in motivational deficits in ADHD patients [37]. For the mesocortical pathway between VTA dopaminergic neurons and the prefrontal cortex, the prefrontal cortex is consistently identified as a region of critical dysfunction in the aetiology of ADHD. For example, hypofrontality, or weakened functioning of the prefrontal cortex, is commonly identified in imaging studies [16]. A decreased absolute number of neurons could potentially contribute to less dopamine release or availability in those target regions. The correlation with a decreased absolute number of dopaminergic neurons in the right VTA is consistent with clinical evidence that dopaminergic deficits are evident in the right cerebrum in human ADHD (e.g., [29,30]).

An effect of perinatal anoxia on VTA dopaminergic neurons has been indirectly implicated [61]. In that study, dopamine hypofunction in the prefrontal cortex was evident in adult rats exposed to birth by caesarean section and 15 min of anoxia. Their results and the current study suggest a neural circuit by which perinatal complications involving low oxygen levels contribute to the aetiology of ADHD.

The new animal model provides a unique opportunity to investigate potential treatments to rescue dopaminergic neurons of the VTA and to prevent ADHD-like hyperactivity and impulsivity in response to delayed reward. Antioxidants are one potential treatment strategy to investigate because dopaminergic neurons are vulnerable to oxidants [62], and oxidants are very likely to be produced during repeated hypoxia. VTA dopaminergic neurons may be specifically vulnerable due to a relative lack of antioxidant defences, increased glutamate receptors, or greater dependence on ATP (i.e., a higher metabolic rate), for example, compared to neighbouring neuronal populations [63]. The specific vulnerability may also be due to an innate differential in expressed protein profiles compared to other midbrain dopaminergic neuronal populations [64]. If a treatment is effective in pre-clinical research, it is proposed that it be administered to extremely premature infants when in a newborn intensive care unit (NICU).

Males born extremely prematurely have a long-term deficit in body weight at adolescence and 20 years of age [65]. We observed a prolonged deficit in body weight in repeated hypoxia male rats, confirming previous results [20,21] and the model’s relevance to extreme prematurity.

### 3.2. Ethical Considerations

The strategy used for the repeated hypoxic exposures was ethically approved (Section 4) [20,21]. The protocol induced apnoea (i.e., a respiratory pause of > 15 s) and was the maximum survivable insult as determined from pilot experiments [20,21]. This strategy was adopted because rats are highly resistant to hypoxia due to their ability to survive in the sewers during floods in the natural environment. After exposure to repeated hypoxia, the mortality rate across all experiments was < 15%, which was ethically approved (Section 4.2) [20,21]. The value of this approach is evident from the spectrum of short- and long-term brain pathology and short- and long-term behavioural deficits in repeated hypoxia male rats, which closely resembles human extreme prematurity [20,21] this study. From PN1 onwards, the rats were regularly weighed to monitor well-being. For the FI-EXT experiments, the animals were food restricted to 85% of their body weight to ensure that they would complete the assigned task, which was based on a food reward [20,21]. This strategy was ethically approved. For the long-term 16-month-old cohort of male rats, they were also food restricted prior to long-term behavioural testing on the FI-EXT task (see Figure 5D in [21]). This approach was used to decrease the risk of foot sores, since some rats had grown to be close to 1 kg in body weight. All of these procedures ensured the welfare and ethical treatment of the animals. All of the experiments were ethically compliant.

### 3.3. Limitations and Future Directions

The open field tests were performed in the same arena (i.e., room) for all tests, but behaviour in a novel environment was an aim. This methodological strategy has been used since the start of open field testing by Hall in 1934 [66] and others (e.g., [67,68,69,70,71]). In our experiment, single (one-day) open field tests were performed on the same animals at 4, 7.5, and 15 months of age (to test for hyperactivity in a novel environment), followed by three sessions on consecutive days when the animals were 18 months of age (to test for hyperactivity in a familiar environment). It is evident that there were major gaps in time between the single sessions, and hence, these were considered novel sessions. This interpretation is consistent with other longitudinal studies in the literature that are cited above. If there was a contribution of familiarization at 7.5 and 15 months of age (i.e., PN221 and PN457, respectively, in Figure 5), then the repeated hypoxic rats would be expected to be hyperactive at these ages on the open field test, but they were not (see Figure 5).

With respect to other technical issues, one of the 16-month-old rats did not meet the criterion for the FI-EXT task [20] and hence was excluded from those data analyses. This context is routine in animal behavioural research. The same rat could be included for the other behavioural and anatomical analyses. For the histology, the data from two 16-month-old rats were excluded due to atypically pale immunostaining. This technical constraint is not unusual in histology.

Anatomical and behavioural correlations in the same rats were completed at 16–18 months of age but not at 3 months of age. No anatomy (i.e., histology) was performed at 3 months, where behavioural changes were shown, because the cerebrum of these rats was used for neurochemical experiments not reported here. The neurochemical experiments required non-perfused tissue. The overall aim of the neurochemical experiments was to correlate the findings with the 3-month-old behavioural results. Since there was dopaminergic neuronal loss in the VTA at PN14 and 18 months of age, it seems likely that this is a lifelong deficit. It is acknowledged that anatomical data at 3 months of age are needed to confirm or refute this.

It Is acknowledged that although the novel animal model may be a useful model of ADHD resulting from extreme prematurity, it may not necessarily reflect ADHD that occurs in the absence of extremely premature birth. It is also acknowledged that extreme prematurity is associated with all three presentations of ADHD and is not just limited to ADHD-HI ([24,72]). Johnson et al. [73] showed that extremely premature children are at a risk of being inattentive, hyperactive, and impulsive, while emphasizing the predominant risk of inattention. This contrasts our findings and those of Oorschot et al. [20], wherein hyperactivity and impulsivity but not inattention were observed in repeated hypoxia rats. For context, the risk of premature birth is higher in mothers who are unemployed and/or received minimal social support [74]. Given that social disadvantage is a risk factor for inattention among premature infants [72,75,76], it is possible that the neurobiological ramifications of an extremely premature birth lead to hyperactivity and impulsivity, whereas psychological stress induced by disadvantaged social context contributes to inattention. In the current study, potential effects of social stressors were controlled for, as repeated hypoxia and repeated normoxia littermates were raised by the same dam. Therefore, the loss of VTA dopaminergic neurons may not be necessary or be sufficient by itself to cause a deficit in attention, which is a multidimensional construct [77]. In further research, a subset of dams could be subjected to psychological stressors and the level of inattention compared between the offspring of dams exposed and not exposed to stress.

Future work using this animal model should investigate its predictive validity (i.e., whether a treatment used on humans with ADHD is effective). This could include the response to methylphenidate and D-amphetamine during testing on the FI task for ADHD-like hyperactivity and ADHD-like impulsivity. Future research could also investigate other relevant behavioural paradigms (e.g., the five-choice serial reaction time task for measuring impulsivity). In addition, future work could investigate the genes most likely to be affected in ADHD [78], the biological basis for the long-term deficit in body weight, the intactness of dopaminergic nerve terminals in the ventral striatum, and the neurochemical changes in the brain. For example, to prove the hypotheses herein at the neurobiochemical level, dopamine levels in the ventral striatum need to be investigated using analytical assays (e.g., electrochemical detection using high-performance liquid chromatography). Future research could also investigate whether there are changes in the circuitry of the prefrontal cortex or changes in cognitive flexibility.

## 4. Materials and Methods

All animal procedures were approved by the Committee on Ethics in the Care and Use of Laboratory Animals at the University of Otago (92/02 EXT, 16/09). Fifty-two male Sprague-Dawley rats (Figure 1) from eight different litters were used (see further details below). Sprague-Dawley rats were used in this study because an FI-EXT paradigm had already been published for rats [42,43]. In addition, the behavioural results could be compared to previous work in the field using the same sub-species [20,21,42]. Rats also produce litters of 8–15 pups, which is efficient for generating enough male rats per group, across 2–3 litters, to achieve statistical power. Rats are also more user-friendly than mice. The sample sizes used have yielded statistical differences in previous behavioural and anatomical studies using this animal model [20,21]. Hence, the study was adequately powered to detect meaningful results.

### 4.1. Repeated Hypoxia and Behaviour: Overview

Postnatal day (PN) 1–3 male rats were exposed to repeated hypoxia or repeated normoxia (the control condition) as previously described [20,21]. They were then tested on an FI-EXT testing schedule of reinforcement in operant chambers from 16 months of age (cohort 1; Oorschot et al. [20]) (Figure 1a) or from PN60 (cohort 2) (Figure 1b). In cohort 1 and in cohort 2, the repeated hypoxia and repeated normoxia rats were from three different litters. One of the repeated normoxia rats did not reach the criterion on the FI-EXT test [20] but was used for other behavioural and anatomical measures, leading to the range of 8–9 (Figure 1a).

FI-EXT schedules of reinforcement have been used to test for hyperactivity, impulsivity, and inattention in ADHD children [7] and in the spontaneously hypertensive rat model of ADHD [7,43]. ADHD-like impulsivity was first investigated in the 16-month-old cohort 1 rats by Oorschot et al. [20] by using recorded data. Using an FI-EXT schedule identical to the one in the current study, our earlier study [20] showed that the same 16-month-old rats were ADHD-like hyperactive but not inattentive (i.e., they were attentive). All three ADHD-like behaviours (i.e., hyperactivity, impulsivity, and inattention) were investigated in the PN60 cohort 2 rats of the current study.

### 4.2. Repeated Hypoxia or Repeated Normoxia

Prior to the repeated hypoxia or repeated normoxia exposure, male offspring were weight-matched on the day of birth (i.e., PN0) and then allocated into either a repeated hypoxia or repeated normoxia group. The randomization into a group within a pair was as follows: For the first pair the heavier pup was the repeated hypoxia pup. For the next pair, the heavier pup was the repeated normoxia pup, and so on for each pair in a litter. This alternating sequence was continued for the other 2–3 litters exposed at the same time. The repeated hypoxia animals were exposed to humidified 1.5% oxygen, 5% carbon dioxide, and 93.5% nitrogen at 37 °C every 2 h for 12.25 h per day from PN1 to 3 [20,21]. This resulted in 7 hypoxic exposures per day, with the first starting at 7 a.m. and the last starting at 7 p.m. Each hypoxic exposure lasted 15 min on PN1 and 14 min on PN2 and PN3. Repeated normoxia pups underwent the same experimental paradigm; however, the exposure was either to humidified air (21% oxygen, balance nitrogen, as in Oorschot et al. [20,21] for the 16-month cohort, and a separate PN14 cohort (see below)) or to normal room air while located on an electric blanket (for the PN60 cohort). This change enabled more pups to be exposed to repeated hypoxia to accommodate a range of different experiments (including some experiments not reported here). This was logistically expedient because pups exposed to repeated hypoxia have a mortality rate (up to 15%, which is ethically approved) compared to no mortality rate for pups exposed to repeated normoxia. This variation for the repeated normoxia pups did not influence the results. For example, a significant increase in ADHD-like hyperactivity with no attention deficit occurred in the repeated hypoxia versus the repeated normoxia rats in the PN60 cohort (this study) and in the older 16-month-old cohort [20]. Hence, ADHD-like hyperactivity with no attention deficit was evident at both ages compared to the respective repeated normoxia rats of the same developmental or adult stage. All pups were returned to their dam in between exposures. Pups were weighed daily from birth until weaning on PN24. Upon weaning, animals were individually housed and continued to be weighed daily until PN35 and during subsequent behavioural testing, and twice weekly at all other times. All animals were housed in a controlled environment on a 12 h light/12 h dark schedule and had access to food and water ad libitum aside from when food was restricted for FI-EXT testing.

### 4.3. FI-EXT Testing of ADHD-like Impulsivity, Hyperactivity, and Inattention

All rats were handled the same for all behavioural analyses. It was not possible to blind the animals during behavioural analyses because the repeated hypoxia animals had decreased body weight (see Section 2.10). Training for the FI-EXT task commenced at 16 months of age (cohort 1) or PN60 (cohort 2). The rationale for the specific ages used is as follows: Cohort 1 rats were trained from 16 months of age because, in characterizing a novel animal model, each new behavioural test (e.g., of motor skills, memory, ADHD-like hyperactivity) needed planning and then time for testing. The logistical timing of each new test also required compatibility between the availability of senior personnel and equipment. Meanwhile, the age of the animals increased (see Figure 5D in Oorschot et al. [21]). In humans, ADHD is prevalent in adulthood (see Section 1). In cohort 2, behavioural training occurred from PN60 because rats can be trained to press a lever in the operant task from this age. Data from cohort 2 were obtained at 3 months of age since it takes up to a month of training and consolidation before the final data for ADHD-like behaviour can be collected from days 23–32 of testing (see further details below).

All rats were food restricted to 85% of their free-feeding weight immediately prior to the start of training. To maintain their body weight at 85%, each animal was given a measured quantity of food daily after testing. Med Associates operant chambers (ENV-008-D1) were used to test rats from PN60. The food reward consisted of sweetened condensed milk delivered into the hopper via a liquid dispenser. All inputs and outputs and data collection were controlled by a computer. Details of the Campden Instruments operant chambers and testing procedure for animals tested at 16 months of age have been described previously [20]. Although different operant chambers were used between the studies at 16 months of age and 2 months of age, all testing and training procedures were identical to those in the prior study [20]. For example, the structural and lighting elements of the operant chambers and the food rewarded were exactly the same for the two cohorts.

Animals were habituated to the operant chambers and trained to access the food hopper and to press the left lever to receive a food reward, as previously described [20]. Specifically, to habituate the animals to the operant chambers, each animal was placed in a chamber with the lights off and levers retracted and allowed to explore for 5 min. Each animal received one session of habituation. The following day further training commenced. The first step was to train each animal to access the food hopper. A computer programme was used that continuously delivered milk to the food hopper every 15 s over a period of 20 min. Every time milk was available the light in the hopper came on. When an animal had reached a criterion of ≥ 30 entries into the hopper during a 20 min session, they were deemed to have reached the criterion and were moved on to the next stage of training. If an animal did not reach the criterion in the first session, they were given additional sessions on successive days until they successfully reached the criterion.

The next stage was to train the animals to press the left lever to receive a food reward. To do this, another computer programme was used that delivered food into the hopper automatically every 30 s as well as when the animal pressed the left lever. A feedback light above the left lever was illuminated whenever the left lever was pressed. During these sessions the right lever was retracted. When each animal had learned to press the left lever to receive food and had reached a criterion of ≥ 150 presses during a 30 min session, they received four further training sessions before moving onto the testing schedule. The animals received one session of training daily.

The FI-EXT testing schedule is a multiple component lights on/lights off schedule (see Figure 1 in Oorschot et al. [20]). We followed the program developed and used by Sagvolden et al. [41] and Sagvolden and Sergeant [42]. During testing, both the left and the right levers were extended. However, only responses on the left lever were rewarded. A response feedback light was present above each lever and lit up when the animal pressed the lever. Each FI period was 2 min long. When a 2 min FI interval had elapsed, the first left lever press thereafter was rewarded. There was no specified time period after each 2 min interval in which the rat had to respond. When a rat responded, the food reward was available for 3 s and then the next FI interval started. During FI periods the house light was on. After the reward was received at the end of the seventh consecutive FI, there was a 5 min EXT component during which the house light was off and no reward was available. After the EXT component, the entire sequence of seven FI periods followed by an EXT period was repeated. Each animal received one session daily for 32 consecutive days. All training and testing sessions were conducted in the rat’s light phase between 1:30 p.m. and 6 p.m.

For FI-EXT testing, only data from the last 10 sessions (i.e., days 23–32) were used for analysis. This is because it takes a number of sessions for stability in the pattern of responding to develop and because ADHD-like behaviour is not normally present in novel situations but develops over time [7,41,42,43,79,80]. The number of lever presses completed during the FI and EXT components was grouped into 10 s bins for the FI periods and 1 min bins for the EXT periods. Data from the first 2 min interval at the beginning of each FI sequence were excluded, as the behaviour of rats during this time period has been found to be remarkably different to their behaviour during the rest of the FI intervals [41,42]. The number of lever presses made in each 10 s or 1 min bin was averaged to calculate an average number of lever presses in each bin for each rat from day 23 to day 32. An ADHD-like hyperactive rat presses the lever more during the last minute of each FI period, which indicates hyperactivity in response to delayed reward [42].

To determine whether the repeated hypoxic rats were impulsive during the FI task, IRTs were distributed into bins of 0.33 s duration across all FI bins from day 23 to day 32 [43] for the repeated hypoxia and repeated normoxia groups. IRTs between left lever presses during FI were allocated into seven inter-response time (IRT) bins (0–0.33 s, 0.34–0.67 s, 0.68–1.00 s, 1.01–1.33 s, 1.34–1.66 s, 1.67–2.00 s, and > 2.00 s, [43]). The IRTs were recorded separately for each 10 s segment of the 2 min FI period. An average number of responses within each bin for each 10 s segment of FI for that day was obtained. The average numbers of responses within each IRT bin for repeated hypoxia and repeated normoxia animals for days 23–32 were then plotted into 3D graphs to show the IRT distribution during the FI component as a function of each 10 s segment. ADHD-like impulsive rats had a higher number of lever presses with short IRTs (e.g., 0–0.33 s, see arrow in Figure 2b,e in Section 2).

The burst index is the difference between the expected frequency, assuming random pressing, and the actual frequency of responses over days 23–32 [42]. A high burst index indicates a preference for responding impulsively with short bursts of activity [42]. To determine the burst index, the expected frequency of responses was calculated using the following formula [42]:

X = −exp [−*t*_1_*p*] + exp [−*t*_2_*p*]

where *p* is the average lever-pressing response rate (per second) across FI, ***t*_1_** is the lower time limit for that bin (e.g., 0.34–0.67 s), and ***t*_2_** is the upper time limit for that bin (e.g., 0.34–0.67 s). The difference between the expected and actual frequencies was calculated and the final burst index was the sum of these differences for each of the three IRT bins containing IRTs less than 1.00 s. After calculating this individually for each rat, the group average of the burst index for repeated hypoxia and repeated normoxia animals was obtained.

### 4.4. Open Field Testing for Hyperactivity

Repeated hypoxia rats were hyperactive in response to delayed reward on the FI-EXT task at 16 months of age [20]. It is unknown whether they are hyperactive on a more general test of locomotor activity, the open field test. Single (novel) or consecutive (familiar) sessions in the open field apparatus were used because ADHD-like hyperactivity is not normally present in novel situations [42,81]. The same rats that were tested on the FI-EXT task at 16 months of age were tested in the open field at several younger ages. Single sessions were carried out at 4, 7.5, and 15 months of age, followed by three sessions on consecutive days at 18 months of age (Figure 1a). The open field apparatus was a 60 cm × 60 cm square field, with 36 equal-sized squares drawn on the floor covering the entire area. Each session was 20 min. Rats were not food restricted during testing. All testing was undertaken during the light phase. Horizontal motor activity was measured by the number of squares entered by each rat with at least two forelimbs. Vertical movements were measured by the number of rearings onto the hindlimbs. The number of times a rat stopped to groom was also counted.

### 4.5. Stereological Analyses of the Absolute Number and Average Somal or Nuclear Volume of Immunostained Dopaminergic Neurons in the Midbrain

On either PN14 or PN545, rats were anaesthetised and intracardially perfused with 4% paraformaldehyde (Agar Scientific, Stansted, Essex, UK) in 0.1 M phosphate buffer (PB). Brains were dissected out, separated into the hemispheres and the hindbrain, and cryoprotected in a 30% sucrose solution in 0.1 M PB. Each brain region was frozen on dry ice and stored at −80 °C. The right hemisphere from each rat was serially sectioned in the sagittal plane into adjacent pairs of 5 µm frozen sections using a cryostat. Four adjacent pairs were systematically collected, after a random start, throughout the right hemisphere onto 3-aminopropylthriethoxy-silane-coated slides. The systematic sampling interval was every 20th section at PN14. At PN545 it was every 20th section for the SNCd and SNCv, and every 40th section for the VTA, CLi, and RRF. Immunohistochemical staining was carried out on one pair of sections (usually the first pair) throughout the right hemisphere. The remaining pairs of sections were stored in a polyethylene glycol antifreeze solution at −20 °C. Some of these pairs were used for a histochemical experiment (detailed below).

For immunohistochemical processing, all steps were completed at room temperature unless otherwise indicated. The sections were washed in 0.1 M phosphate buffered saline (PBS, 3 × 5 min) and incubated in 5% heat-inactivated goat serum (Sigma-Aldrich, St Louis, Missouri, USA) in PBS for 1 h to block non-specific binding sites. The sections were then incubated in a polyclonal rabbit anti-TH antibody (Chemicon, Katy, Texas, USA, 1:48,000) in antibody-diluting buffer (1% bovine serum albumin (Sigma-Aldrich, A-2153, St Louis, Missouri, USA) with 0.1% Tween-20 in 0.1 M PBS) overnight at 4 °C. Immunohistochemical processing using this TH antibody has previously been found to accurately stain for TH-positive dopaminergic neurons within the rat SNCd [82]. TH is also expressed by norepinephrine neurons, but these neurons are located within the brainstem and not within the midbrain [32]. Sections were then washed in 0.1 M PBS (3 × 5 min), followed by incubation in a biotinylated goat anti-rabbit secondary antibody (Vector Laboratories Inc., Burlingame, CA, USA, 1:200) in antibody-diluting buffer with 5% goat serum for 1 h. After washing in 0.1 M PBS (3 × 5 min), the sections were incubated in hydrogen peroxide (0.3% in 0.1 M PBS for 10 min) to inactivate endogenous peroxidases. Sections were then washed in 0.1 M PBS (3 × 5 min), followed by incubation in a streptavidin-conjugated horseradish peroxidase tertiary antibody (Vector Laboratories, Burlingame, CA, USA, 1:200). The antigen/antibody complex in the sections was then visualised with 0.014% 3-amino-9-ethyl-carbazole (AEC, Sigma-Aldrich, St Louis, Missouri, USA) and 0.3% hydrogen peroxide in acetate buffer. The sections were then coverslipped in glycerol gelatine (Sigma-Aldrich, St Louis, Missouri, USA) for subsequent stereological analyses. Negative control sections with the primary antibody omitted were included in all experiments to confirm that the staining was specific to the TH neurons.

All sections were coded prior to stereological analyses. The absolute (i.e., total) number of TH-positive dopaminergic neurons in the right VTA, CLi (at PN545), SNCd, SNCv, and RRF was measured using Cavalieri’s method to estimate the absolute reference volume (Vref) and the (physical) disector method [46,56,83] to measure the N_v_ of dopaminergic neurons in pairs of adjacent 5 µm sections. The physical disector method is used when other stereological methods, such as the optical disector method, are unable to be performed due to minimal penetration of antibodies through thicker 50 µm sections of the PN14 or PN545 brain. Some of the stereological methods used were not computerized, yet they are still valid practical approaches [52]. It is acknowledged that computerized approaches are more common now (e.g., [84]).

One efficient way to perform both the Vref and physical disector methods was to take high-power photographs of the midbrain nuclei in each 5 µm section that was stained. This allowed for easy and accurate matching of neurons between disector pairs. Photographs of each section were taken using the ×10 objective of an Olympus AX70 light microscope (Tokyo, Japan). Using this objective, two to three images were usually required to capture the entire structure in each section. The final magnification of the images was ×220. Once the images of each structure were taken for each section, the images were printed, aligned, and sellotaped together to make one large montage of each section. This made it easier to identify common features between pairs of sections.

To apply Cavalieri’s method to measure the Vref, one section (i.e., printed montage) from each pair was used. Using previously defined criteria ([52,85,86]; Figure 5a–d and the text below), the boundaries of the right VTA, CLi, SNCd, SNCv, and RRF were drawn onto the printed montages. In the most lateral sections, there was a thin dense band of TH staining superior or dorsal to the cerebral peduncle that was identified as the start of the SNCd. In more medial sections, the band of SNCd neurons lengthened and became more diffuse posteriorly (Figure 5a). When this band shortened anteriorly and became double the height of the lateral SNCd it was defined as the starting point of the VTA (Figure 5c). At this point, there was also an obvious extension of the staining trailing in the posterior and superior direction, beginning in the last few sections of the SNCd and continuing into the VTA. This was the RRF (Figure 5c). As the SNCd progressed from lateral to medial, there was also a posterior cluster of TH-positive neurons in the substantia nigra pars reticulata (SNR). This posterior cluster was identified as part of the SNCv (Figure 5b) that extends into the SNR. This ventral tier of SNC dopaminergic neurons was present in the sections until two to three sections after the first appearance of the VTA. In more lateral sections, the posterior cluster of SNCv neurons was separated from the SNCd, RRF, and VTA by non-immunostained gaps (Figure 5a–c). In sections where the RRF and SNCv clusters were not clearly separated by a non-immunostained gap, the SNCv cluster was more densely stained than the RRF. This permitted their borders to be delineated.

The VTA began laterally as a widening and shortening of the SNCd (Figure 5c), as described above. In the section after the VTA first appeared, it was separated into two parts by the medial terminal nucleus (MT) of the accessory optic tract for the following two to three sections (Figure 5c). The oculomotor nerve was usually visible in the VTA at two or three sections before the most medial section. The fasciculus retroflexus appeared anteriorly to the VTA one or two sections before the most medial section, usually after the appearance of the oculomotor nerve. In the most medial sections, the VTA was at its largest and changed shape to become more circular (Figure 5d). In the most medial sections, the dopaminergic neurons of the VTA extended superiorly and posteriorly into the CLi. Although the CLi is part of the dorsal raphe group, it is considered one of the individual nuclei of the VTA, as it is continuous with the other VTA nuclei and is predominantly dopaminergic. At PN545, the CLi and VTA nuclei were separately identified and the absolute number of neurons was quantified for each part. At PN14, the CLi was harder to identify and hence was not separately identified from the VTA.

The RRF usually began in the last one to two sections containing the SNCd or the first section containing the VTA (Figure 5b,c). It was an obvious band of staining extending superiorly and caudally to the VTA. In one to two sections prior to the end of the RRF, the VTA and RRF were separated by a non-immunostained gap, the medial lemniscus (ML) (Figure 5d). When the ML was not present and there was no staining extending superiorly, the RRF had ended and only the VTA remained. The RRF usually ended two or three sections before the most medial section.

Once the borders were drawn around each midbrain region, a specific grid on an acetate sheet was used with points spaced apart at the following distances, PN14, VTA: 25 × 25 mm, RRF, SNCv: 15 × 15 mm, SNCd: 10 × 10 mm; 18 months old, all: 30 × 30 mm. This grid was overlaid randomly on the printed images of each respective structure. Then the number of points landing on each dopaminergic region for each of the sections was counted and summed for the entire right hemisphere (∑P). The following formula [87] was used to calculate the final Vref of each dopaminergic region:

Vref = ∑P ^.^ a(p) ^.^ *t* ^.^ s

where the section thickness (*t*) in this case was 0.005 mm and the section interval sampled (s) was 20 at PN14 and 20 or 40 at PN545. The area of each point, a(p), was calculated by dividing the length between each grid point by the magnification of the image and squaring it.

The physical disector method was used to calculate the average number of TH-positive neurons in a known subvolume of each midbrain region (i.e., the N_v_). A transparent plastic acetate sheet with square sampling frames measuring 30 × 30 mm for the SNCd at PN14, 20 × 20 mm for the SNCd at PN545, and 50 × 50 mm for the other nuclei was overlaid on the image of each of the nuclei on one of the sections from each pair. This section was referred to as the reference section. At PN14, a sampling interval of every square for the SNCd and every second square for the other structures was used. AT PN545, a sampling interval of every second square for the VTA and every square for the other structures was used. According to this sampling interval, the square frames that landed with at least half of their area over the structure of interest were drawn on the image. Once all the sampling frames had been drawn on each section, a transparent plastic acetate sheet was overlaid on top of each structure. The outline of the nucleus and the square grids to be sampled were then traced onto the acetate sheet. Any identifying features of the image, such as blood vessels or obvious nuclei, were also traced onto the acetate sheet. Once this was completed, the traced image from the first section of the pair on the acetate sheet was lined up and overlaid on top of the image of the second section from that pair. As each section was only 5 µm apart, it was usually easy to use the identifying features and the shape of the midbrain region to match up the first section to the identical position in the second section from the pair. The same sampling frames were then drawn on the second section. The second section was referred to as the look-up section. Each grid to be sampled was numbered for identification.

All of the individual TH-positive nuclei in each sampling frame of the reference section were then drawn onto the acetate sheets or drawn using Adobe Photoshop version 11 (Adobe Inc., San Jose, CA, USA) on a computer-captured image of the section and were marked with a tick if the TH-positive neuron had a nucleus and a cross if the TH-positive neuron did not have a nucleus [46]. Once each frame was completed for the first section, the same acetate sheet or computerized drawing was lined up over the look-up section from that pair and the sample grids were matched up to the same area on that image. The TH-positive neurons from the reference section were then matched with the same neurons in the look-up section. The neurons that were present in both sections were then marked on the acetate or computerized drawing with another tick if the nucleus was still present in the look-up section and a cross if the nucleus was not present in the look-up section. The neuron was also counted if its nucleus was present in the look-up section but not in the reference section. The number of neurons within each sampling frame for each individual midbrain region that had a visible nucleus in one section but not the other section was recorded for each pair of sections. These neurons were termed “disector neurons” (Q^−^). The entire procedure was repeated on each pair of sections throughout the right hemisphere for each midbrain dopaminergic structure.

To yield the absolute number of disector neurons (Q^−^) for each midbrain structure, the counts from each pair of sections were summed. The absolute number of TH-positive neurons within each midbrain dopaminergic nucleus was calculated by first using the following formula to calculate the total sampled volume (TSV) or total disector volume:

TSV (mm^3^) = ∑F ^.^ ASF ^.^ *t* (1)

where ∑F = the total number of frames counted, ASF = the area of the sampling frame = (size mm/magnification of image)^2^, and *t* = section thickness. The Vref and TSV were then used to calculate the absolute number of TH-positive neurons in each right midbrain structure for each rat using the following formula:

Vref/TSV ^.^ ∑Q^−^ (2)

where ∑Q^−^ = total number of disector neurons counted. The data were then uncoded and the average number of TH-positive neurons in each midbrain structure was calculated for each group and age. Due to atypically pale immunostaining, the data from two repeated normoxia animals were excluded, yielding data from 7 of 9 repeated normoxia animals in cohort 1 (Figure 1a).

### 4.6. Histochemical and Stereological Analyses of the Absolute Number of Midbrain Neurons

Pairs of sections immediately adjacent to the sections that were sampled for TH staining were selected from a subgroup of the brains at PN14 and PN545 (n = 3 per group and n = 7–8 per group, respectively). These sections were stained with 0.1% filtered thionin-acetate (Sigma, St Louis, Missouri, USA, T7029), dehydrated through a series of alcohol rinses, mounted with DPX (dibutylphthalate polystyrene xylene), and coded. Images were taken at a magnification of ×220, printed, and montaged together. The identifying features from the TH sections were matched to the corresponding features on the thionin section to enable the borders to be drawn around the midbrain region of interest. Stereological analyses were then undertaken using Cavalieri’s method and the physical disector technique as described above. This yielded the absolute number of all neurons within the VTA, SNCd, SNCv, and RRF of PN14 repeated normoxia versus repeated hypoxia rats. At PN545, the absolute number of all neurons within the VTA, VTA + Cli, and SNCd of repeated normoxia versus repeated hypoxia rats was obtained.

### 4.7. Stereological Measurement of the Average Somal Volume of Midbrain Dopaminergic Neurons

The point-sampled linear intercepts method [56,88] was used to measure the volume-weighted somal or nuclear volume of the TH-positive neurons. After a random start, every second sampling frame throughout the entire VTA and every sampling frame in the SNCd was viewed using a 100× oil-immersion objective lens (numerical aperture 1.4) on an Olympus BH-2 light microscope. An automated electronic stage was fitted onto the microscope and a video camera projected the image onto an adjacent television monitor. Overlaying the television monitor was an unbiased sampling frame with sine-weighted angles and measuring 19 cm × 19 cm. At each sampled frame, a linear intercept grid composed of parallel lines with evenly spaced intercept points (see Figure 6 in Mayhew [88]) was placed over the frame and aligned to the particular sine-weighted angle that was generated from a random number table. If an intercept point fell on the soma of a TH-positive neuron within the unbiased sampling frame, a ruler was used to measure the distance from one edge of the soma to the opposite edge of the soma in the direction of the linear grid (see Figure 6 in Mayhew [88]). At PN545, the distance from one edge to the opposite edge of the nucleus was measured. If more than one intercept point fell on the same neuron, then all lines containing an intercept point were measured and the average length of those lines was recorded for that neuron. The sum of all the intercept lengths from all neurons in the midbrain structure was then divided by the number of neurons sampled to yield the average length for a rat. This average in mm was then divided by the magnification to yield the average in µm, termed “l.” The volume-weighted (Vv) of the soma or nucleus for each rat was calculated from:

Vv = π/3 ^.^ l^3^ µm^3^. (3)

The somal or nuclear volume for each rat was then averaged for the repeated normoxia versus repeated hypoxia group and statistically compared.

### 4.8. Coefficient of Error of the Stereological Estimates

The CE was calculated based on Gundersen et al. [53] for the absolute Vref and the absolute number of neurons within the midbrain structures analysed. The CE of the numerical density (i.e., N_V_ or number per subvolume) estimate of the dopaminergic neurons within the midbrain structures analysed was calculated as 1/(∑Q^−^)^1/2^ [89]. For the mean somal volume of midbrain dopaminergic neurons, the CE was calculated as CE = SEM (ln(*v*)) = CV/(∑Q^−^)^1/2^, where *v* is the individual neuronal somal estimates for each rat and CV is the coefficient of variation of these estimates within each rat [90].

### 4.9. Statistical Analyses

Averages were expressed as mean ± SEM. An ANOVA for repeated measures was used to statistically compare FI and EXT responding, IRT distributions, body weight data, and between-session analyses on the open field task. Bonferroni’s post-hoc analyses were completed for the open field test, with correction for multiple comparisons. No correction for multiple comparisons was needed for the other analyses since post-hoc comparisons were not undertaken. For the stereological results and the burst index, the data for each measured parameter were first checked for the normality of the distribution. If the criteria for normality were met, a Student’s *t*-test was performed. If the criteria were not met, a Mann–Whitney *U* test was performed. Possible associations between the behavioural and anatomical data of the 16–18-month-old rats were analysed using Pearson’s correlation (corrected for multiple comparisons). *p* ≤ 0.05 was considered statistically significant. All statistical analyses were completed using Prism v9.3.1.

## 5. Conclusions

Since 1775, we have learned much about ADHD, and yet we are only beginning to understand how changes in the brain contribute to the disorder [57]. The findings of this paper have established a new animal model of the hyperactive/impulsive presentation of ADHD. The hyperactivity and the impulsivity were detected after delayed reward, a hallmark of ADHD. The findings also enable investigations of potential new treatments that prevent the loss of VTA dopaminergic neurons and the development of the hyperactive-impulsive subtype/presentation of ADHD.

The theoretical implications of the research are that ADHD-like hyperactivity and ADHD-like impulsivity, specifically in response to delayed reward, should be a primary component of the design of future experiments that characterize potential animal models of ADHD. Based on the results, it is recommended that the testing of hyperactivity in an open field apparatus should not be the primary and only measure of hyperactivity. Another theoretical implication of the research is that the new animal model will enable future investigations of the biology of the hyperactive/impulsive presentation of ADHD, including analysis of the circuitry of the prefrontal cortex and of cognitive flexibility.

The significance statement for this research is as follows: Prevention of ADHD and improvements in medication are significantly hindered by a lack of accurate animal models, particularly of the hyperactive/impulsive presentation. We investigated a novel rat model. Males exposed to repeated hypoxia during the equivalent of extreme prematurity exhibited ADHD-like hyperactivity and ADHD-like impulsivity in response to delayed reward, with no attention deficit. In the short and long term, repeated hypoxia rats had a specific significant loss of dopaminergic neurons in the right VTA. Fewer VTA dopaminergic neurons correlated with increased ADHD-like hyperactivity and impulsivity. Intervention therapies at birth, novel to ADHD, can now be investigated to rescue VTA dopaminergic neurons and potentially prevent ADHD-like hyperactivity/impulsivity. A permanent cure could ensue.

## Figures and Tables

**Table 1:** Stereological data for the dopaminergic neurons in the VTA for the repeated normoxia versus repeated hypoxia rats at PN14 and PN545.

Measured Parameter	Number of Rats	Vref (mm^3^) of VTA	N_v_ (per mm^3^) of Dopaminergic Neurons in the VTA	*N,* Dopaminergic Neurons in the VTA
**Experimental Condition**				
**VTA dopaminergic**				
**neurons, PN14**				
*Repeated normoxia*	6			
Mean		0.682	17,034	11,479
SD		0.114	1689	1042
SEM		0.051	755	466
*Repeated hypoxia*	8			
Mean		0.585 *	15,269 **	8894 ***
SD		0.058	1855	1060
SEM		0.022	701	400
**VTA dopaminergic**				
**neurons, PN545**				
*Repeated normoxia*	7			
Mean		0.989	13,277	12,798
SD		0.145	2970	1682
SEM		0.059	1213	687
*Repeated hypoxia*	10			
Mean		1.007 †	11,163 ††	11,213 †††
SD		0.148	1830	2125
SEM		0.049	610	708

* 14% decrease, *p* = 0.0696, two-tailed Student’s *t*-test; NS, not statistically significant. ** 10% decrease, *p* = 0.0926, two-tailed Student’s *t*-test; NS. *** 23% decrease, *p* = 0.0007, two-tailed Student’s *t*-test. † 2% increase, *p* = 0.8059; NS. †† 16% decrease, *p* = 0.0953, two-tailed Student’s *t*-test; NS. ††† 12% decrease, *p* = 0.0439, one-tailed Student’s *t*-test. *N*, absolute number of neurons; Nv, numerical density; PN, postnatal day; Vref, total reference volume; VTA, ventral tegmental area.

**Table 2:** Final mean coefficient of error (CE) values of useful parameters from the right midbrain of repeated normoxia and repeated normoxia rats based on the formula CE = (1/n. ∑i CE_i_^2^)^1/2^.

Measured Parameter	CE Vref *	CE N_v_ **	CE *N* ***
Experimental Condition †			
**Absolute number of VTA dopaminergic**			
**neurons, PN14**			
Repeated normoxia	0.013	0.037	0.039
Repeated hypoxia	0.014	0.044	0.045
**Absolute number of SNCd dopaminergic**			
**neurons, PN14**			
Repeated normoxia	0.018	0.069	0.071
Repeated hypoxia	0.018	0.071	0.074
**Absolute number of SNCv dopaminergic**			
**neurons, PN14**			
Repeated normoxia	0.021	0.101	0.102
Repeated hypoxia	0.021	0.1	0.101
**Absolute number of RRF dopaminergic**			
**neurons, PN14**			
Repeated normoxia	0.014	0.078	0.079
Repeated hypoxia	0.014	0.08	0.081
**Absolute number of VTA dopaminergic**			
**neurons, PN545**			
Repeated normoxia	0.017	0.06	0.061
Repeated hypoxia	0.018	0.062	0.063
**Absolute number of VTA + CLi dopaminergic**			
**neurons, PN545**			
Repeated normoxia	0.017	0.054	0.054
Repeated hypoxia	0.017	0.056	0.058
**Absolute number of SNCd dopaminergic**			
**neurons, PN545**			
Repeated normoxia	0.052	0.087	0.09
Repeated hypoxia	0.05	0.081	0.082
**Absolute number of SNCv dopaminergic**			
**neurons, PN545**			
Repeated normoxia	0.034	0.097	0.099
Repeated hypoxia	0.027	0.094	0.096
**Absolute number of RRF dopaminergic**			
**neurons, PN545**			
Repeated normoxia	0.031	0.117	0.117
Repeated hypoxia	0.029	0.122	0.127
**Absolute number of VTA thionin-**			
**stained neurons, PN14**			
Repeated normoxia	0.012	0.035	0.04
Repeated hypoxia	0.014	0.041	0.043
**Absolute number of SNCd thionin-**			
**stained neurons, PN14**			
Repeated normoxia	0.018	0.056	0.059
Repeated hypoxia	0.017	0.06	0.063
**Absolute number of SNCv thionin-**			
**stained neurons, PN14**			
Repeated normoxia	0.019	0.075	0.077
Repeated hypoxia	0.018	0.064	0.065
**Absolute number of RRF thionin-**			
**stained neurons, PN14**			
Repeated normoxia	0.014	0.048	0.049
Repeated hypoxia	0.013	0.049	0.051
**Absolute number of VTA thionin-**			
**stained neurons, PN545**			
Repeated normoxia	0.022	0.049	0.051
Repeated hypoxia	0.023	0.051	0.052
**Absolute number of VTA + CLi thionin-**			
**stained neurons, PN545**			
Repeated normoxia	0.018	0.044	0.045
Repeated hypoxia	0.018	0.045	0.046
**Absolute number of SNCd thionin-**			
**stained neurons, PN545**			
Repeated normoxia	0.059	0.072	0.076
Repeated hypoxia	0.056	0.065	0.067

† To measure the absolute number of dopaminergic (i.e., tyrosine hydroxylase-positive) or thionin-stained neurons, an average of 6 to 15 sections were analysed in each midbrain structure for each stain per rat. The respective averages were *PN14*, VTA 15, SNCd 6, SNCv 9, RRF 13; *PN545*, VTA 11, VTA + CLi 11, SNCd 8, SNCv 7, RRF 10. This generally fulfilled the guideline of sampling 8–10 sections per brain structure per animal (Korbo et al. [55]). The average of 6–7 analysed sections for the smaller SNCd and SNCv resulted from the adoption of a strategy during serial sectioning that permitted efficient systematic sampling of sections across all the midbrain structures of interest in each animal. * The average for the total number of points counted to estimate the absolute reference volume (Vref) at PN14 ranged from 226 to 583 across the four midbrain structures and the two experimental conditions. This range is for the sampled sections stained with either tyrosine hydroxylase or thionin. This average exceeded the guideline of counting 100 points per structure (Gundersen et al. [53]). For the averages at PN545 the range was 105 to 362 for the RRF, SNCv, VTA, and VTA + Cli. It was 60–63 for the SNCd. The real area of the counted points was altered at PN14 to accommodate the relative size of each structure, including the smaller SNCd. This was not the case at PN545 and led to the lower average point count for the SNCd. The resulting CE Vref for the SNCd at PN545 ranged from 0.050 to 0.052 (see table above), and this was less than the biological variability (CV, see Table 3 and Table 4), indicating that reliable measurements were obtained. ** The average number of dopaminergic or thionin-stained neurons sampled per disector volume ranged from 0.65 to 10.21 for the midbrain structures analysed in the two experimental conditions at PN14 and PN545. The average number generally exceeded or complied with the guideline of sampling 1–2 cells per disector volume (West and Gundersen [51]). Specifically, the respective average number of thionin-stained neurons counted per disector volume in the two experimental conditions ranged from 2.54 to 10.21 for all the midbrain structures at PN14 and PN545. The respective average number of dopaminergic neurons counted per disector volume in the two experimental conditions ranged from 2.98 to 4.34 for all the midbrain structures at PN14 and from 1.11 to 3.32 for the VTA, VTA + CLi, SNCv, and RRF at PN545. For the SNCd at PN545 it was 0.65–0.66. The resulting CE for Nv and *N* for the SNCd at PN545 ranged from 0.081 to 0.090 (see table above), and this was less than the biological variability (CV, see Table 3 and Table 4), indicating that reliable measurements were obtained. *** The average total number of disector neurons sampled per structure ranged from 78 to 804 for the midbrain structures in the two experimental conditions at PN14 and PN545. This range is for the sampled sections stained with either tyrosine hydroxylase or thionin. These averages mainly fulfilled or exceeded the recommendation of counting 100–200 disector neurons per brain structure per animal (Gundersen et al. [56]). Specifically, for the averages at PN14, the range was 100 to 804 for the four midbrain structures. For the averages at PN545, the range was 116 to 540 for all midbrain structures except the RRF. It was 78–81 for the disector dopaminergic neurons in the RRF. The resulting CE for Nv and *N* for these neurons in the RRF at PN545 ranged from 0.117 to 0.127 (see table above). This was less than the biological variability (CV, see Table 2) for both *N* and one Nv, indicating that reliable measurements were obtained. The other Nv had a low CV of 10.2%. In this situation, the CE of 0.122 is deemed acceptable (Slomianka and West [54]).

**Table 3:** Summary of the precision of the stereological measurements for the midbrain dopaminergic neurons.

Measured Parameter	(Mean CE)^2^	(CV)^2^ *	(Mean CE)^2^ < 1/2 (CV)^2^?
**Experimental Condition** **Absolute number of VTA dopaminergic neurons, PN14**			
Repeated normoxia, Vref	(0.013)^2^	(0.168)^2^	Yes
Repeated hypoxia, Vref	(0.014)^2^	(0.098)^2^	Yes
Repeated normoxia, Nv	(0.037)^2^	(0.099)^2^	Yes
Repeated hypoxia, Nv	(0.044)^2^	(0.121)^2^	Yes
Repeated normoxia, *N*	(0.039)^2^	(0.091)^2^	Yes
Repeated hypoxia, *N*	(0.045)^2^	(0.119)^2^	Yes
**Absolute number of SNCd dopaminergic neurons, PN14**			
Repeated normoxia, Vref	(0.018)^2^	(0.139)^2^	Yes
Repeated hypoxia, Vref	(0.018)^2^	(0.106)^2^	Yes
Repeated normoxia, Nv	(0.069)^2^	(0.129)^2^	Yes
Repeated hypoxia, Nv	(0.071)^2^	(0.143)^2^	Yes
Repeated normoxia, *N*	(0.071)^2^	(0.220)^2^	Yes
Repeated hypoxia, *N*	(0.074)^2^	(0.148)^2^	Yes
**Absolute number of SNCv dopaminergic neurons, PN14**			
Repeated normoxia, Vref	(0.021)^2^	(0.092)^2^	Yes
Repeated hypoxia, Vref	(0.021)^2^	(0.153)^2^	Yes
Repeated normoxia, Nv	(0.101)^2^	(0.199)^2^	Yes
Repeated hypoxia, Nv	(0.100)^2^	(0.191)^2^	Yes
Repeated normoxia, *N*	(0.102)^2^	(0.169)^2^	Yes
Repeated hypoxia, *N*	(0.101)^2^	(0.204)^2^	Yes
**Absolute number of RRF dopaminergic neurons, PN14**			
Repeated normoxia, Vref	(0.014)^2^	(0.181)^2^	Yes
Repeated hypoxia, Vref	(0.014)^2^	(0.123)^2^	Yes
Repeated normoxia, Nv	(0.078)^2^	(0.151)^2^	Yes
Repeated hypoxia, Nv	(0.080)^2^	(0.144)^2^	Yes
Repeated normoxia, *N*	(0.079)^2^	(0.263)^2^	Yes
Repeated hypoxia, *N*	(0.081)^2^	(0.172)^2^	Yes
**Absolute number of VTA dopaminergic neurons, PN545**			
Repeated normoxia, Vref	(0.017)^2^	(0.146)^2^	Yes
Repeated hypoxia, Vref	(0.018)^2^	(0.147)^2^	Yes
Repeated normoxia, Nv	(0.060)^2^	(0.225)^2^	Yes
Repeated hypoxia, Nv	(0.062)^2^	(0.164)^2^	Yes
Repeated normoxia, *N*	(0.061)^2^	(0.131)^2^	Yes
Repeated hypoxia, *N*	(0.063)^2^	(0.190)^2^	Yes
**Absolute number of VTA + CLi dopaminergic neurons, PN545**			
Repeated normoxia, Vref	(0.017)^2^	(0.208)^2^	Yes
Repeated hypoxia, Vref	(0.017)^2^	(0.154)^2^	Yes
Repeated normoxia, Nv	(0.054)^2^	(0.201)^2^	Yes
Repeated hypoxia, Nv	(0.056)^2^	(0.174)^2^	Yes
Repeated normoxia, *N*	(0.054)^2^	(0.108)^2^	Yes
Repeated hypoxia, *N*	(0.058)^2^	(0.182)^2^	Yes
**Absolute number of SNCd dopaminergic neurons, PN545**			
Repeated normoxia, Vref	(0.052)^2^	(0.185)^2^	Yes
Repeated hypoxia, Vref	(0.050)^2^	(0.158)^2^	Yes
Repeated normoxia, Nv	(0.087)^2^	(0.139)^2^	Yes
Repeated hypoxia, Nv	(0.081)^2^	(0.234)^2^	Yes
Repeated normoxia, *N*	(0.090)^2^	(0.269)^2^	Yes
Repeated hypoxia, *N*	(0.082)^2^	(0.221)^2^	Yes
**Absolute number of SNCv dopaminergic neurons, PN545**			
Repeated normoxia, Vref	(0.034)^2^	(0.153)^2^	Yes
Repeated hypoxia, Vref	(0.027)^2^	(0.111)^2^	Yes
Repeated normoxia, Nv	(0.097)^2^	(0.222)^2^	Yes
Repeated hypoxia, Nv	(0.094)^2^	(0.197)^2^	Yes
Repeated normoxia, *N*	(0.099)^2^	(0.123)^2^	No, 12.3% CV is low
Repeated hypoxia, *N*	(0.096)^2^	(0.134)^2^	No, 13.4% CV is low
**Absolute number of RRF dopaminergic neurons, PN545**			
Repeated normoxia, Vref	(0.031)^2^	(0.205)^2^	Yes
Repeated hypoxia, Vref	(0.029)^2^	(0.288)^2^	Yes
Repeated normoxia, Nv	(0.117)^2^	(0.210)^2^	Yes
Repeated hypoxia, Nv	(0.122)^2^	(0.102)^2^	No, 10.2% CV is low
Repeated normoxia, *N*	(0.117)^2^	(0.265)^2^	Yes
Repeated hypoxia, *N*	(0.127)^2^	(0.295)^2^	Yes
**Somal volume of VTA dopaminergic neurons, PN14**			
Repeated normoxia, ln(*v*)	(0.057)^2^	(0.249)^2^	Yes
Repeated hypoxia, ln(*v*)	(0.056)^2^	(0.121)^2^	Yes
**Somal volume of SNCd dopaminergic neurons, PN14**			
Repeated normoxia, ln(*v*)	(0.086)^2^	(0.153)^2^	Yes
Repeated hypoxia, ln(*v*)	(0.085)^2^	(0.183)^2^	Yes
**Nuclear volume of VTA dopaminergic neurons, PN545**			
Repeated normoxia, ln(*v*)	(0.097)^2^	(0.284)^2^	Yes
Repeated hypoxia, ln(*v*)	(0.117)^2^	(0.192)^2^	Yes

* CV = standard deviation of the mean/mean.

**Table 4:** Summary of the precision of the stereological measurements for the midbrain neurons histochemically stained with thionin.

Measured Parameter	(Mean CE)^2^	(CV)^2^ *	(Mean CE)^2^ < 1/2 (CV)^2^?
**Experimental Condition** **Absolute number of VTA thionin-stained neurons, PN14**			
Repeated normoxia, Vref	(0.012)^2^	(0.146)^2^	Yes
Repeated hypoxia, Vref	(0.014)^2^	(0.059)^2^	Yes
Repeated hormoxia, Nv	(0.035)^2^	(0.037)^2^	No, 3.7% CV is low ***
Repeated hypoxia, Nv	(0.041)^2^	(0.080)^2^	Yes
Repeated normoxia, *N*	(0.040)^2^	(0.136)^2^	Yes
Repeated hypoxia, *N*	(0.043)^2^	(0.119)^2^	Yes
**Absolute number of SNCd thionin-stained neurons, PN14**			
Repeated normoxia, Vref	(0.018)^2^	(0.034)^2^	Yes
Repeated hypoxia, Vref	(0.017)^2^	(0.036)^2^	Yes
Repeated normoxia, Nv	(0.056)^2^	(0.082)^2^	Yes
Repeated hypoxia, Nv	(0.060)^2^	(0.196)^2^	Yes
Repeated normoxia, *N*	(0.059)^2^	(0.241)^2^	Yes
Repeated hypoxia, *N*	(0.063)^2^	(0.232)^2^	Yes
**Absolute number of SNCv thionin-stained neurons, PN14**			
Repeated normoxia, Vref	(0.019)^2^	(0.206)^2^	Yes
Repeated hypoxia, Vref	(0.018)^2^	(0.110)^2^	Yes
Repeated normoxia, Nv	(0.075)^2^	(0.002)^2^	No, 0.2% CV is low
Repeated hypoxia, Nv	(0.064)^2^	(0.117)^2^	Yes
Repeated normoxia, *N*	(0.077)^2^	(0.122^2^	Yes
Repeated hypoxia, *N*	(0.065)^2^	(0.012)^2^	No, 1.2% CV is low
**Absolute number of RRF thionin-stained neurons, PN14**			
Repeated normoxia, Vref	(0.014)^2^	(0.198)^2^	Yes
Repeated hypoxia, Vref	(0.013)^2^	(0.149)^2^	Yes
Repeated normoxia, Nv	(0.048)^2^	(0.236)^2^	Yes
Repeated hypoxia, Nv	(0.049)^2^	(0.053)^2^	No, 5.3% CV is low
Repeated normoxia, *N*	(0.049)^2^	(0.270)^2^	Yes
Repeated hypoxia, *N*	(0.051)^2^	(0.159)^2^	Yes
**Absolute number of VTA thionin-stained neurons, PN545**			
Repeated normoxia, Vref	(0.022)^2^	(0.079)^2^	Yes
Repeated hypoxia, Vref	(0.023)^2^	(0.141)^2^	Yes
Repeated normoxia, Nv	(0.049)^2^	(0.095)^2^	Yes
Repeated hypoxia, Nv	(0.051)^2^	(0.113)^2^	Yes
Repeated normoxia, *N*	(0.051)^2^	(0.118)^2^	Yes
Repeated hypoxia, *N*	(0.052)^2^	(0.214)^2^	Yes
**Absolute number of VTA + CLi thionin-stained neurons, PN545**			
Repeated normoxia, Vref	(0.018)^2^	(0.222)^2^	Yes
Repeated hypoxia, Vref	(0.018)^2^	(0.187)^2^	Yes
Repeated normoxia, Nv	(0.044)^2^	(0.112)^2^	Yes
Repeated hypoxia, Nv	(0.045)^2^	(0.124)^2^	Yes
Repeated normoxia, *N*	(0.045)^2^	(0.123)^2^	Yes
Repeated hypoxia, *N*	(0.046)^2^	(0.195)^2^	Yes
**Absolute number of SNCd thionin-stained neurons, PN545**			
Repeated normoxia, Vref	(0.059)^2^	(0.371)^2^	Yes
Repeated hypoxia, Vref	(0.056)^2^	(0.168)^2^	Yes
Repeated normoxia, Nv	(0.072)^2^	(0.474)^2^	Yes
Repeated hypoxia, Nv	(0.065)^2^	(0.218)^2^	Yes
Repeated normoxia, *N*	(0.076)^2^	(0.178)^2^	Yes
Repeated hypoxia, *N*	(0.067)^2^	(0.260)^2^	Yes

* CV = standard deviation of the mean/mean.

## Data Availability

The data presented in this study are available on request from the corresponding author.
